# Interface Engineering of Co‐LDH@MOF Heterojunction in Highly Stable and Efficient Oxygen Evolution Reaction

**DOI:** 10.1002/advs.202002631

**Published:** 2020-11-25

**Authors:** Zhenxing Li, Xin Zhang, Yikun Kang, Cheng Cheng Yu, Yangyang Wen, Mingliang Hu, Dong Meng, Weiyu Song, Yang Yang

**Affiliations:** ^1^ State Key Laboratory of Heavy Oil Processing College of New Energy and Materials China University of Petroleum (Beijing) Beijing 102249 China; ^2^ College of Science China University of Petroleum (Beijing) Beijing 102249 China; ^3^ Department of Materials Science and Engineering California Nano Systems Institute University of California Los Angeles CA 90095 USA

**Keywords:** density functional theory, interface engineering, layered double hydroxide, metal–organic frameworks, oxygen evolution reaction

## Abstract

The electrochemical splitting of water into hydrogen and oxygen is considered one of the most promising approaches to generate clean and sustainable energy. However, the low efficiency of the oxygen evolution reaction (OER) acts as a bottleneck in the water splitting process. Herein, interface engineering heterojunctions between ZIF‐67 and layered double hydroxide (LDH) are designed to enhance the catalytic activity of the OER and the stability of Co‐LDH. The interface is built by the oxygen (O) of Co‐LDH and nitrogen (N) of the 2‐methylimidazole ligand in ZIF‐67, which modulates the local electronic structure of the catalytic active site. Density functional theory calculations demonstrate that the interfacial interaction can enhance the strength of the Co—O_out_ bond in Co‐LDH, which makes it easier to break the H‐O_out_ bond and results in a lower free energy change in the potential‐determining step at the heterointerface in the OER process. Therefore, the Co‐LDH@ZIF‐67 exhibits superior OER activity with a low overpotential of 187 mV at a current density of 10 mA cm^−2^ and long‐term electrochemical stability for more than 50 h. This finding provides a design direction for improving the catalytic activity of OER.

## Graphical abstract

The interface of Co‐LDH@ZIF‐67 is built by the oxygen of cobalt layered double hydroxide (Co‐LDH) and nitrogen of the ZIF‐67, which can modulate the electronic structure of the catalytical site. The Co‐LDH@ZIF‐67 exhibits superior catalytical activity in oxygen evolution reaction with a low overpotential of 187 mV at a current density of 10 mA cm^−2^.

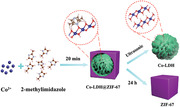

The consumption of fossil fuels and the increase of environmental issue limit the global economic growth. The development of an eco‐friendly strategy for the production of efficient and sustainable energy could fundamentally settle the issues related to resource, energy, and environment.^[^ ^1^ ^]^ Driven by the exploration of clean and sustainable energy, the electrochemical splitting water into hydrogen and oxygen has been considered as one of the most promising approaches.^[^ ^2^, ^3^, ^4^ ^]^ Since it is a thermodynamically unfavorable process, the oxygen evolution reaction (OER) plays a key role in the process of water splitting. Moreover, the low efficiency of OER is the bottleneck of water decomposition. In order to address the problem mentioned above, it is critical to develop highly efficient and stable electrocatalysts for the OER.

Although previous studies have confirmed that some noble metal‐based oxide nanomaterials such as IrO_2_ and RuO_2_ show high electrochemical activity for OER,^[^ ^5^, ^6^ ^]^ the scarcity and high cost of these materials severely restrict their large‐scale industrial use. Therefore, developing efficient, low‐cost, and stable catalysts for OER has significant scientific and research value. 2D ultrathin nanosheets with significant lateral dimensions can offer more catalytically active sites.^[^ ^7^, ^8^ ^]^ Recently, layered double hydroxides (LDHs) have attracted increasing attention owing to their special morphologies, high catalytic activity in OER, and facile synthesis methods.^[^ ^9^, ^10^ ^]^ An increasing number of studies have indicated that Co‐based electrocatalysts can effectively promote the oxygen catalytic reaction in alkaline electrolytes.^[^ ^11^, ^12^, ^13^ ^]^ In particular, Co‐based LDH is regarded as one of the most efficient OER catalysts. In previous studies, Co‐based LDH showed high OER catalytic activity. However, the stability of these materials remains to be improved.^[^ ^14^, ^15^ ^]^ The Co—O bond in Co‐based LDH is the main catalytically active site in OER. Therefore, an effective way to improve the catalytic performance of Co‐based LDH is to modulate the local electronic structure of the Co—O bond. ZIF‐67, a type of Co‐based metal–organic framework (MOF), the organic ligand of 2‐methylimidazole, is a credible candidate for this purpose.^[^ ^16^ ^]^ It has uniform pore diameter distribution and ultrahigh surface area, making it a feasible choice for endowing the electrocatalyst with the structural advantages of large surface areas and reasonably distributed pores to improve its catalytic performance.^[^ ^17^, ^18^, ^19^, ^20^ ^]^

We designed a novel Co‐LDH@ZIF‐67 core–shell heterojunction structure to enhance the catalytic activity and stability of Co‐LDH. The interfacial interaction between ZIF‐67 and Co‐LDH, large surface area, and porous structure led to the high catalytic activity and stability of OER. We simply deposited Co‐LDH onto ZIF‐67 to prepare Co‐LDH/ZIF‐67. Compared with pure Co‐LDH, Co‐LDH/ZIF‐67 showed lower electrochemical performance. Therefore, the excellent OER activity of Co‐LDH@ZIF‐67 can be attributed to its unique structure and the heterojunction interface, which was built by the oxygen (O) of Co‐LDH and nitrogen (N) of the 2‐methylimidazole ligand in ZIF‐67. The formation of the interface modulated the local electronic structure of the catalytic active site. Density functional theory (DFT) calculations demonstrated that the interfacial interaction enhanced the strength of the Co—O_out_ bond in Co‐LDH, which made it easier to break the H—O_out_ bond and resulted in a lower free energy change in the potential‐determining step (PDS) in OER. This finding provides a new direction to improving the catalytic activity of OER.

The well‐shaped Co‐LDH@ZIF‐67 with a core–shell heterojunction structure was prepared via a simple aqueous solution‐based method using 2‐methylimidazole as the ligand and Co(NO_3_)_2_ as the cobalt precursor (**Scheme** **1**). This special architecture was obtained after 20 min of reaction at 25 °C. To the best of our knowledge, this is the first time that an intermediate structure of Co‐LDH@ZIF‐67 has been obtained in the synthesis process of ZIF‐67. As depicted in Figure S2, Supporting Information, the hollow structure of ZIF‐67 gradually disappeared when the reaction time increased. When the reaction time was increased to 24 h, ZIF‐67 was obtained. It is speculated that Co‐LDH nucleated rapidly when 2‐methylimidazole solution was poured into Co(NO_3_)_2_ solution. Then ZIF‐67 gradually grew around, forming Co‐LDH@ZIF‐67. With the increase of the reaction time, 2‐methylimidazole gradually seized Co from Co‐LDH, and finally formed a complete ZIF‐67. Note that Co‐LDH@ZIF‐67 can also be converted into ultrathin Co‐LDH nanosheets (1.5 nm) by the ultrasonic process in the aqueous solution with extremely low concentration of 0.015 mg mL^−1^ (Figure S3, Supporting Information). Co‐LDH/ZIF‐67 was obtained by adding 2‐methylimidazole to the Co‐LDH aqueous solution and reacting at 25 °C for 6 h (Figure S4a, Supporting Information).

**Scheme 1:**
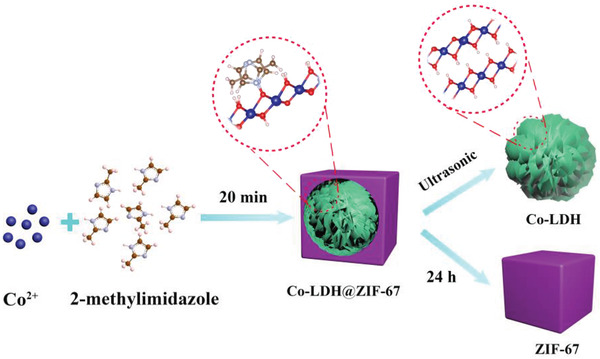
Schematic illustration of growth pathway to prepare Co‐LDH@ZIF‐67, Co‐LDH, and ZIF‐67.

The morphology of Co‐LDH@ZIF‐67 was examined by scanning electron microscopy (SEM) (**Figure** **1**a,b; Figure S1, Supporting Information). Co‐LDH@ZIF‐67 has a cubic morphology and is highly monodisperse, with a particle size of 350 nm (Figure 1a). High‐magnification SEM (Figure 1b) shows that Co‐LDH@ZIF‐67 has a hollow structure and Co‐LDH is encapsulated in this structure. A schematic of Co‐LDH@ZIF‐67 is shown in Figure 1c. Transmission electron microscopy (TEM) showed that the Co‐LDH encapsulated in ZIF‐67 was an ultrathin nanosheet (Figure 1d,e) grown on the internal surface of ZIF‐67. The high‐resolution TEM (HRTEM) image clearly shows the interface of the heterojunction between Co‐LDH and ZIF‐67 (Figure 1f; Figure S7, Supporting Information). The lattice fringe with a d‐spacing of 0.275 nm is consistent with the (100) diffraction plane of Co‐LDH, and the d‐spacings of 0.346 nm are attributed to the (224) diffraction planes of ZIF‐67.

**Figure 1:**
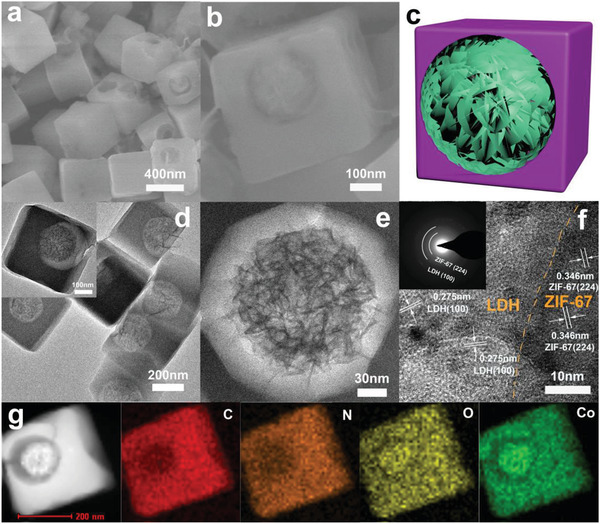
a,b) SEM image of Co‐LDH@ZIF‐67. c) Schematic of Co‐LDH@ZIF‐67. d,e) TEM image of Co‐LDH@ZIF‐67. f) HRTEM image of Co‐LDH@ZIF‐67. g) EDX elemental mappings of Co‐LDH@ZIF‐67.

The selected area electron diffraction (SAED) pattern of Co‐LDH@ZIF‐67 can be well indexed to the crystal planes of LDH and MOF (inset to Figure 1f). The diffraction rings correspond to the (224) diffraction planes of ZIF‐67 and (100) diffraction planes of Co‐LDH, further confirming the heterojunctions between Co‐LDH and ZIF‐67. To further investigate the Co‐LDH@ZIF‐67 heterojunction structure, scanning transmission electron microscopy (STEM) was used to examine a Co‐LDH@ZIF‐67 nanoparticle. High‐angle annular dark‐field scanning transmission electron microscopy (HAADF‐STEM) and energy‐dispersive X‐ray spectroscopy (EDS) elemental mapping (Figure 1g) clearly demonstrated that C, N, O, and Co were uniformly distributed in the whole shell,^[^ ^21^, ^22^ ^]^ whereas C and N were not present in the core portion. These results strongly suggest the successful fabrication of the core–shell heterojunction structure of Co‐LDH@ZIF‐67. The as‐obtained ZIF‐67 was a nanocube, with an average edge length of 350 nm (Figure S2b, Supporting Information). The hollow structure was not observed in ZIF‐67.

The SEM results showed that the morphology and shape of Co‐LDH were derived from Co‐LDH@ZIF‐67 by the ultrasonic process within the aqueous solution (**Figure** **2**a). Uniform nanosheets of Co‐LDH were assembled into nanosheet clusters. The architecture of Co‐LDH is clearly observed in Figure 2b,c. The nanosheet clusters had flower‐like morphologies composed of many nanosheets with slight wrinkles. The Co‐LDH nanosheets were ultrathin, which caused slight wrinkles. Atomic force microscopy (AFM) results (Figure 2e,f) demonstrated that the average thickness of the Co‐LDH nanosheets was 1.5 nm. This ultrathin structure generated a significant specific surface area, which led to high catalytic activity for OER. The HRTEM image illustrates the well‐resolved lattice spacings with an interplane distance of 0.275 nm, in accordance with the (100) plane of Co‐LDH (Figure 2d). Meanwhile, the HAADF‐STEM‐EDS elemental mapping (Figure 2h) showed that both Co and O atoms were homogeneously distributed throughout the nanosheet clusters, which was consistent with the HRTEM result.

**Figure 2:**
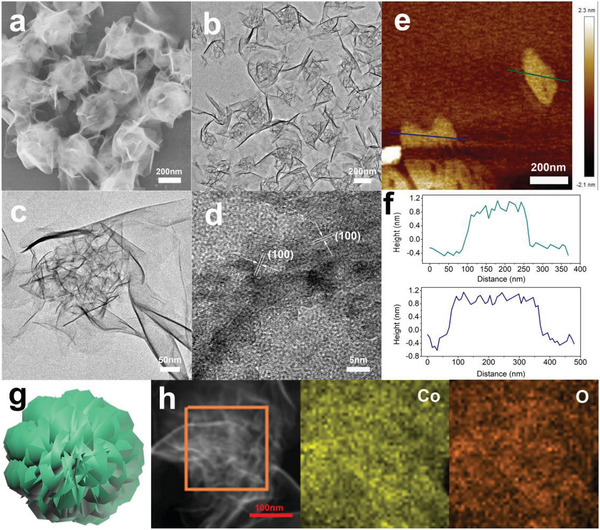
a) SEM image of Co‐LDH. b,c) TEM images of Co‐LDH. d) HRTEM image of Co‐LDH. e,f) AFM images and the corresponding thickness measurement data of Co‐LDH. g) Schematic of edge‐cut Co‐LDH. h) EDX elemental mappings of Co‐LDH.

The as‐obtained Co‐LDH@ZIF‐67 core–shell structure can be further explained by X‐ray diffraction (XRD). The XRD patterns of Co‐LDH, Co‐LDH@ZIF‐67, and ZIF‐67 are presented in **Figure** **3**a and that of Co‐LDH/ZIF‐67 is shown in Figure S4b, Supporting Information. The XRD pattern of ZIF‐67 displayed diffraction peaks at 7.4°, 10.4°, 12.7°, 16.5°, and 18.1°, corresponding to the (011), (002), (112), (013), and (222) planes, which was in accordance with the simulated results of ZIF‐67. As for Co‐LDH, all diffraction peaks could be well indexed to Co‐LDH (JCPDS no. 30‐0443),^[^ ^23^ ^]^ and a dominant peak at 19.1° was characteristic of the (001) plane. For the XRD patterns of Co‐LDH@ZIF‐67 and Co‐LDH/ZIF‐67, both the diffraction peaks of ZIF‐67 and Co‐LDH were observed. The diffraction peaks at 7.4°, 10.4°, 12.7°, 16.5°, and 18.1° corresponded to the (011), (002), (112), (013), and (222) planes of ZIF‐67, and the diffraction peaks at 19.1° corresponded to the (001) plane of Co‐LDH, which indicated that Co‐LDH@ZIF‐67 and Co‐LDH/ZIF‐67 consisted of Co‐LDH and ZIF‐67. Similar results were obtained from the thermogravimetric analysis (TGA) under argon atmosphere; the TGA profiles of Co‐LDH, Co‐LDH@ZIF‐67, and ZIF‐67 are shown in Figure 3b. TGA profile of ZIF‐67, similar to the previously reported,^[^ ^24^, ^25^ ^]^ exhibited a small weight loss before 350 °C, owning to the escape of residual solvent from the filler pores. Up to 500 °C, the profile showed a significant drop, suggesting a large amount of weight loss, which can be attributed to the collapse of the crystal structure of ZIF‐67. While the TGA profile of Co‐LDH only revealed a weight loss at about 200 °C, and no more obviously weight loss before 800 °C. The Co‐LDH@ZIF‐67 followed three‐step degradation process, and first weight loss that occurred at 200 °C was the Co‐LDH. Second weight loss occurred at 200–350 °C was ZIF‐67 and last weight loss temperature occurred at 500 °C is lightly lower than ZIF‐67, because of the hollow structure of Co‐LDH@ZIF‐67. To further study the ratio of Co‐LDH and ZIF‐67 in Co‐LDH@ZIF‐67, the TGA of Co‐LDH, Co‐LDH@ZIF‐67, and ZIF‐67 was also taken under air atmosphere. According to the weight loss of pure Co‐LDH and pure ZIF‐67 in the TGA data, the weight formula is used to roughly estimate the amount of Co‐LDH in the composite material to be about 24.71%.

**Figure 3:**
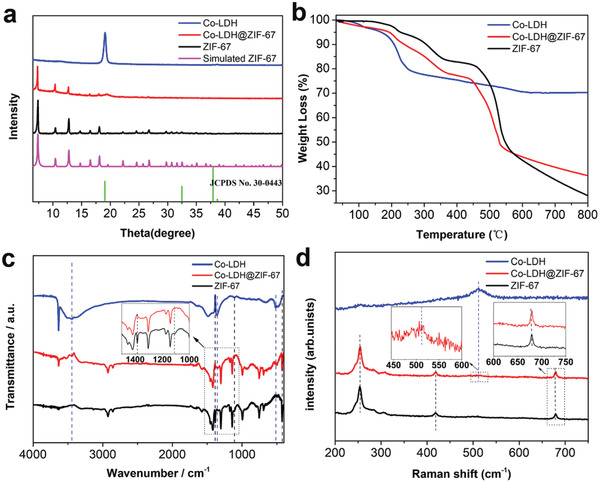
a) XRD pattern (green line: JCPDS no. 30‐0443). b) TGA curve under argon atmosphere. c) FTIR spectrum, and d) Raman spectra of Co‐LDH@ZIF‐67, Co‐LDH, and ZIF‐67.

To further investigate the interface interaction between ZIF‐67 and Co‐LDH, the Fourier transform infrared spectroscopy (FTIR) spectra of Co‐LDH, Co‐LDH@ZIF‐67, and ZIF‐67 were obtained, as shown in Figure 3c; Figure S6a, Supporting Information. For ZIF‐67, the peak at 428 cm was indexed to the Co—N stretch mode of ZIF‐67, and the peaks at 689 and 755 cm were indexed to the out‐of‐plane bending mode of 2‐methylimidazole. The peaks at 1109, 1139, and 1173 cm were attributed to the in‐plane bending of the imidazole ring. The peak at 1306 cm was ascribed to the N—H bending mode of 2‐methylimidazole, and the peaks at 1382, 1417, and 1453 cm can be ascribed to the C—N stretch mode. In addition, the peak at 2960 cm was designated as —CH3.^[^ ^26^, ^27^, ^28^, ^29^, ^30^ ^]^ The peak of Co‐LDH at 516 cm was attributed to the stretching vibration of Co—OH, whereas the peak at 1356 cm was related to the N—O stretching vibration of the anion of the nitrate inserted into the layer.^[^ ^31^, ^32^ ^]^ As expected, the vibrational peak of Co‐LDH@ZIF‐67 consisted of those of Co‐LDH and pure ZIF‐67. When compared with ZIF‐67, the peak at 1382 cm of the C—N stretch mode was not observed, and the peak at 1109 cm of the in‐plane bending mode of the imidazole ring was weakened and even disappeared because of the interfacial effect between Co‐LDH and ZIF‐67.^[^ ^33^, ^34^, ^35^ ^]^The interaction between the O of Co‐LDH and N of the imidazole ring led to the disappearance of the low‐frequency C—N stretch and the weakening of the low‐frequency in‐plane bending mode of the imidazole ring.^[^ ^36^, ^37^ ^]^ Similarly, the Raman spectra of Co‐LDH@ZIF‐67 (Figure 3d; FigureS6b, Supporting Information) contained several typical peaks from 200 to 700 cm corresponding to ZIF‐67 and Co‐LDH, respectively. The peak at 512.0 cm was ascribed to the Co—O stretching of Co‐LDH,^[^ ^38^ ^]^ and the peaks at 251.7, 415.2, and 681.0 cm were ascribed to ZIF‐67.^[^ ^39^ ^]^ Among them, the Co—N bond at 415.2 cm was in good agreement with that of ZIF‐67. Interestingly, the vibrational mode of 2‐methylimidazolate showed an apparent blueshift from 677.6 cm (ZIF‐67) to 681.0 cm (Co‐LDH@ZIF‐67) owing to the electronic effect between Co‐LDH and 2‐methylimidazolate of ZIF‐67, which agreed well with the FTIR results.^[^ ^40^, ^41^ ^]^

X‐ray photoelectron spectroscopy (XPS) is an effective tool to study the composition and chemical state of surface elements. XPS results confirmed the interface interaction in Co‐LDH@ZIF‐67. The full XPS spectra of Co‐LDH@ZIF‐67, Co‐LDH, and ZIF‐67 shown in **Figure** **4**a demonstrated the existence of C, N, O, and Co in Co‐LDH@ZIF‐67 and ZIF‐67, whereas Co‐LDH had no peaks corresponding to N. The Co 2p XPS spectrum of Co‐LDH@ZIF‐67 (Figure 4b) showed two sets of doublet peaks corresponding to Co^2+^ (798.3 and 782.8 eV) and Co^3+^ (796.7 and 780.8 eV) together with two satellite peaks of 803.1 and 786.8 eV.^[^ ^42^ ^]^ In addition, peaks corresponding to Co^3+^ were not observed for ZIF‐67 (Figure S5f, Supporting Information). In this way, the existence of Co^3+^ can be ascribed to the generation of Co‐LDH (Figure S5d, Supporting Information). As depicted in Figure S8, Supporting Information, the UV–vis spectrum of Co‐LDH@ZIF‐67 displayed two absorption peaks. The peak at 598 nm could be indexed to Co(II) ions in tetrahedral environments.^[^ ^43^ ^]^ Moreover, the weak peak at 426 nm could be unambiguously ascribed to the distorted tetrahedron coordination Co(III),^[^ ^44^ ^]^ which was in good agreement with the XPS results. The O 1s XPS spectrum of Co‐LDH@ZIF‐67 (Figure 4d), Co‐LDH (Figure S5g, Supporting Information), and ZIF‐67 (Figure S5i, Supporting Information) could be deconvoluted into two peaks centered at 531.4 and 533.2 eV, which were attributed to the —OH and oxygen configuration in water molecules, respectively. The N 1s spectrum (Figure 4c) showed two peaks, and the most intense peak centered at the binding energy of 398.9 eV. These were assigned to pyridinic N. Note that the peak at the binding energy of 400.5 eV corresponds to pyridine‐*N*‐oxide,^[^ ^45^ ^]^ confirming that the N of the imidazole ring was bonded to the O of Co‐LDH in Co‐LDH@ZIF‐67. Compared with Co‐LDH@ZIF‐67, there is no peak related to pyridine‐*N*‐oxide in the N 1s spectrum of ZIF‐67 (Figure S5c, Supporting Information), instead there was a peak at 400.8 eV, which belonged to pyrrole N.^[^ ^46^ ^]^ This agrees well with the FTIR and Raman results.

**Figure 4:**
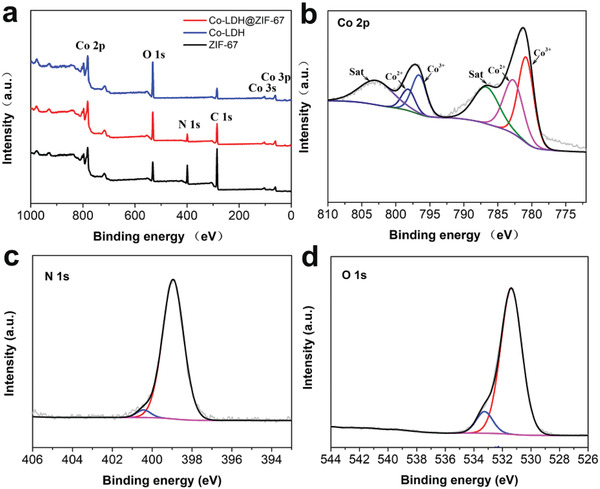
a) XPS wide‐scan spectra of Co‐LDH@ZIF‐67, Co‐LDH, and ZIF‐67. b) High‐resolution Co 2p XPS spectrum. c) High‐resolution N 1s XPS spectrum and d) high‐resolution O 1s XPS spectrum of the Co‐LDH@ZIF‐67.

The porosities and surface areas of ZIF‐67, Co‐LDH@ZIF‐67, and Co‐LDH were analyzed by N_2_ adsorption–desorption isotherm measurements (**Figure** **5**). The specific surface area of Co‐LDH@ZIF‐67 (422.1 m^2^ g^−1^) was significantly higher than that of Co‐LDH (63.4 m^2^ g^−1^), indicating that the core–shell structure can significantly increase the surface area of Co‐LDH. Therefore, such a characteristic structure can provide a high number of catalytically active sites and facilitate the inflow and outflow of reactants, products, and electrolytes effectively. The specific surface area of Co‐LDH@ZIF‐67 was slightly smaller than that of ZIF‐67 (583.6 m^2^ g^−1^) because of the formation of a core–shell structure. In addition, both Co‐LDH@ZIF‐67 and ZIF‐67 had narrow pore size distributions around 1.01 nm.

**Figure 5:**
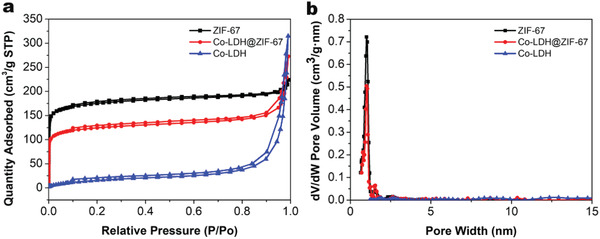
a) N_2_ adsorption–desorption isotherms for Co‐LDH@ZIF‐67, Co‐LDH, and ZIF‐67 and b) pore size distribution plot for Co‐LDH@ZIF‐67, Co‐LDH, and ZIF‐67.

The electrocatalytic OER performance of Co‐LDH@ZIF‐67 was evaluated using a typical three‐electrode setup in a 1 m KOH aqueous solution.^[^ ^47^, ^48^, ^49^, ^50^ ^]^ The electrocatalytic OER activities of Co‐LDH, ZIF‐67, Co‐LDH/ZIF‐67, and IrO_2_ were also investigated for comparison. The linear sweep voltammetry (LSV) of all samples was measured with 80% iR compensation^[^ ^51^ ^]^ (**Figure** **6**a). Co‐LDH@ZIF‐67 showed the lowest onset overpotential toward OER (144 mA) among the four catalysts. The overpotentials at 10 and 30 mA cm^−2^ are summarized in Figure 6b. Co‐LDH@ZIF‐67 presented low overpotentials with values of 187 and 262 mV at current densities of 10 and 30 mA cm^−2^ in 1 m KOH, which were obviously more negative than those of Co‐LDH (194 and 280 mV), IrO_2_ (227 and 303 mV), Co‐LDH/ZIF‐67 (246 and 326 mV), and ZIF‐67 (320 and 375 mV), respectively. Compared with Co‐LDH@ZIF‐67, carbon cloth has almost no OER activity (Figure S9, Supporting Information). It should be mentioned that the overpotential at 30 mA cm^−2^ of Co‐LDH@ZIF‐67 was lower than most electrode materials reported recently, such as Co_3_O_4_/CeO_2_ nanohybrids,^[^ ^52^ ^]^ (Ni_2_Co_1_)_0.925_Fe_0.075_‐MOF‐NF,^[^ ^53^ ^]^ Co–Fe double‐atom catalyst,^[^ ^54^ ^]^ and PM‐LDH^[^ ^55^ ^]^ (Table S1, Supporting Information). Compared with Co‐LDH and Co‐LDH/ZIF‐67, Co‐LDH@ZIF‐67 suddenly enhanced the OER catalytic activity because of the interface effects between Co‐LDH and ZIF‐67. The Tafel slope is defined as *η* = *a* + *b *× log[*j*] (where *η* is the overpotential, *a* is the exchange current density, *b* is the Tafel slope, *j* is the measured current density). The Tafel slope is a useful parameter for describing the kinetic of OER.^[^ ^56^, ^57^, ^58^ ^]^ The Tafel value for Co‐LDH@ZIF‐67 was 59 mV dec^−1^, which was significantly lower than those of IrO_2_ (78 mV dec^−1^), Co‐LDH (83 mV dec^−1^), Co‐LDH/ZIF‐67 (102 mV dec^−1^), and ZIF‐67 (106 mV dec^−1^), suggesting the higher kinetics during OER (see Figure 6c).

**Figure 6:**
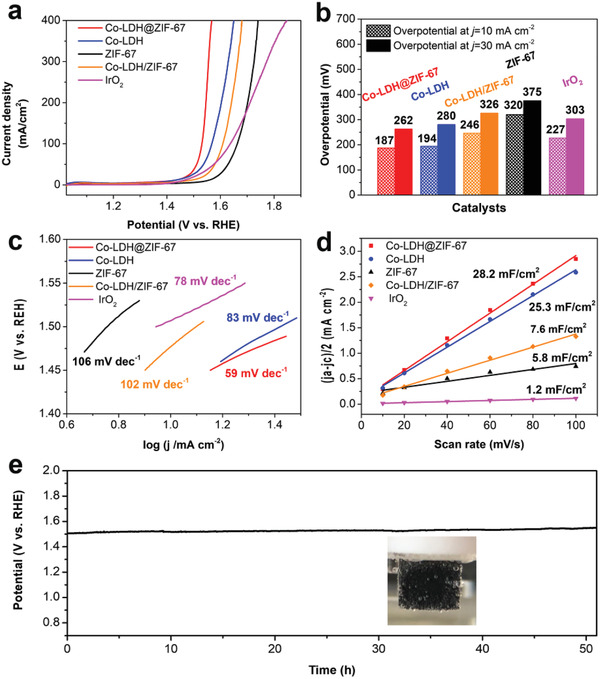
Electrochemical properties of Co‐LDH@ZIF‐67, Co‐LDH, ZIF‐67, Co‐LDH/ZIF‐67, and IrO_2_ for OER. a) OER polarization curve. b) Comparison of onset overpotentials and overpotentials at 30 mA cm^−2^. c) The corresponding Tafel plots derived from (a). d) Plots used to extract the double‐layer capacitances (*C*_dl_) and estimate the relative electrochemically active surface areas. e) Long‐term stability test of Co‐LDH@ZIF‐67 carried out under a constant current density of 30 mA cm^−2^ (inset: the production of O_2_ bubbles on the carbon cloth with Co‐LDH@ZIF‐67 as the electrode).

The electrochemical surface area (ECSA) is an essential parameter for investigating the electrode interface kinetics of electrocatalysts. We obtained ECSAs of the designed heterojunctions by cyclic voltammetry (CV) in the 1.124–1.324 V range (Figure S10a–e, Supporting Information). Double‐layer capacitances (*C*_dl_) were measured to evaluate the ECSAs of Co‐LDH@ZIF‐67, Co‐LDH, ZIF‐67, and IrO_2_. As shown in Figure 6d, the *C*_dl_ of Co‐ LDH@ZIF‐67 (28.2 mF cm^−2^) was larger than those of Co‐LDH (25.3 mF cm^−2^), Co‐LDH/ZIF‐67 (7.6 mF cm^−2^), ZIF‐67 (5.8 mF cm^−2^), and IrO_2_ (1.2 mF cm^−2^), indicating that Co‐LDH@ZIF‐67 exposed more catalytically active sites for OER. Electrochemical stability is an essential parameter for comprehensively assessing the OER performance of an electrocatalyst. The stabilities of Co‐LDH@ZIF‐67, Co‐LDH, ZIF‐67, Co‐LDH/ZIF‐67, and IrO_2_ were evaluated using a typical three‐electrode setup in 1 m KOH. The results are shown in Figure 6e; Figures S11–S14, Supporting Information. It can be seen that the Co‐LDH@ZIF‐67 electrocatalyst maintained the current density of 30 mA cm^−2^ for a minimum of 50 h, with an increment of 8.83% in its overpotential. In comparison, the Co‐LDH, ZIF‐67, Co‐LDH/ZIF‐67, and IrO_2_ electrocatalysts showed increases of 32.60%, 16.31%, 18.75%, and 10.10% in the required overpotential at a density of 30 mA cm^−2^ for 24 h, respectively. Co‐LDH@ZIF‐67 not only exhibited higher OER activity but also excellent stability.

To further investigate the morphology and structure changes of Co‐LDH@ZIF‐67 after OER process, SEM, TEM, HAADF‐STEM‐EDS mapping images, XRD, Raman, and XPS were measured, as shown in Figures S15 and S16, Supporting Information. The SEM and TEM images of Co‐LDH@ZIF‐67 after OER process are shown in Figure S15a,b, Supporting Information, which showed that the morphology of Co‐LDH@ZIF‐67 still maintained core–shell structure. It can be seen from the HAADF‐STEM‐EDS mapping image (Figure S15c, Supporting Information) that there is no N in the core portion, which further confirms that the Co‐LDH@ZIF‐67 still maintained the core–shell structure after electrochemical testing. For the XRD pattern of Co‐LDH@ZIF‐67 after OER process (Figure S15d, Supporting Information), the diffraction peaks of ZIF‐67 and Co‐LDH are observed. However, the diffraction peak of ZIF‐67 was partially weakened. This indicates that Co‐LDH@ZIF‐67 was still composed of Co‐LDH and ZIF‐67 with part of the ZIF‐67 shell destroyed. Similarly, the XPS results showed that the peak positions of Co, C, N, and O did not change (Figure S16, Supporting Information), confirming the presence of the interface in Co‐LDH@ZIF‐67 after OER process. However, the peak area of Co^3+^ in the Co 2p XPS spectrum of Co‐LDH@ZIF‐67 has increased. This can be attributed to the oxidation of the ZIF‐67 shell. This is strongly consistent with the XRD results. The Raman spectrum of Co‐LDH@ZIF‐67 after OER process (Figure S15e, Supporting Information) was not changed, which can be considered that the interface between Co‐LDH and ZIF‐67 still existed. Therefore, the OER process has a slight effect on the morphology of Co‐LDH@ZIF‐67 without destroying its core–shell heterojunction structure. In order to test the stability of Co‐LDH@ZIF‐67 in KOH solution, the low‐magnification SEM image of Co‐LDH@ZIF‐67 deposited on the working electrode in 1 m KOH solution for 24 h is shown in Figure S17, Supporting Information. The morphology of Co‐LDH@ZIF‐67 still maintained, which strongly suggested the stability of ZIF‐67@Co‐LDH in KOH solution.

In addition, electrochemical impedance spectroscopy (EIS) was used to investigate the reaction kinetics and interfacial electron transfer of Co‐LDH@ZIF‐67, Co‐LDH, ZIF‐67, Co‐LDH/ZIF‐67, and IrO_2_ (Figure S10f, Supporting Information). All EIS data were fitted with the same equivalent circuit model (inset to Figure S10f, Supporting Information). According to the fitted EIS results (Table S3, Supporting Information), the R2 of Co‐LDH@ZIF‐67 is the smallest (7.336 Ω), indicating rapid charge transfer kinetics, which was consistent with the results of the LSV test. The low OER activity of Co‐LDH/ZIF‐67 indicated the importance of the heterojunction interface for Co‐LDH@ZIF‐67. Because of the interaction between the well‐interfaced Co‐LDH and ZIF‐67, Co‐LDH@ZIF‐67 exhibited prominent activity and stability in OER.

To further understand the superior activity and stability of the OER of Co‐LDH@ZIF‐67, DFT calculations were performed to reveal the origin of the interfacial effect in OER. Considering that Co‐LDH was grown in ZIF‐67, we established a model of Co‐LDH@ZIF‐67 with the upper and lower layers, representing the 2‐methylimidazole ligand of the inner ZIF‐67 and outer Co‐LDH, respectively (**Figure** **7**b; Figure S18b,d, Supporting Information). As shown in Figure 7b, the N in the 2‐methylimidazole was bonded to the inner O (denoted as O_in_) of Co‐LDH, whereas the outer O (denoted as O_out_) was responsible for the catalytic active site of OER. To further demonstrate the interfacial effect of ZIF‐67 on Co‐LDH and verify the trend in the OER activity in our experiment, we also established a two‐layer Co(OH)_2_ model without 2‐methylimidazole ligand to simulate Co‐LDH for comparison (Figure 7a, **Table** **1**; Figure S18a,c, Supporting Information). Based on the signal peak of Co^3+^ observed in the XPS spectrum and to make a comparison on the same starting line, we investigated the dehydrogenation surface for both models, and take the surface with only one hydrogen as the unified starting point (Figures S19 and S20, Supporting Information). As can be seen from the calculated four‐step OER free‐energy diagrams for Co‐LDH (Figure 7c) and Co‐LDH@ZIF‐67 (Figure 7d),^[^ ^59^, ^60^ ^]^ the PDS of Co‐LDH was dehydrogenation to form the adsorbed oxygen intermediate (OH*→O*), which was the same as that of Co‐LDH@ZIF‐67. In addition, the free‐energy change in the PDS of Co‐LDH@ZIF‐67 (1.94 eV) was significantly lower than that of Co‐LDH (2.16 eV), indicating a better OER performance of Co‐LDH@ZIF‐67 than that of Co‐LDH, which is consistent with the trend in the OER activity in the experiment.

**Figure 7:**
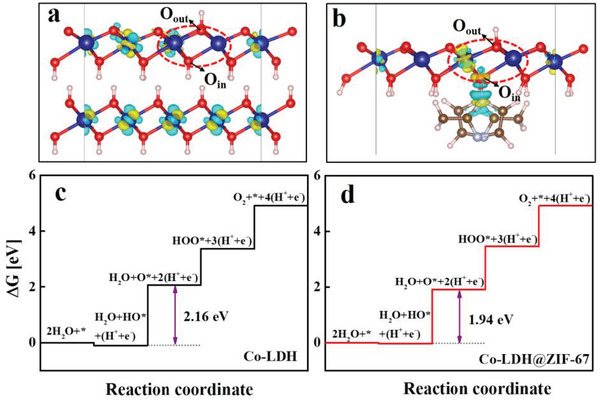
The charge density difference of the underlying layer for a) Co‐LDH and b) Co‐LDH@ZIF‐67, and the Gibbs free energy diagram for OER on c) Co‐LDH and d) Co‐LDH@ZIF‐67. Yellow (blue) isosurfaces denote an increase (decrease) of 0.015 e Å^−3^ for electronic density.

For an in‐depth investigation of the electron transfer and Co—O bond strength in Co‐LDH@ZIF‐67, we calculated projected crystal orbital Hamilton population (pCOHP) to reveal the interaction of cobalt atoms (where Co_1_ was taken as the research object) with O_in_ and O_out_, respectively (**Figure** **8**).^[^ ^61^ ^]^ Moreover, we computed the integrated COHP (ICOHP) by calculating the energy integral up to the Fermi level for each investigated Co—O bond to compare the bond strength quantitatively (**Table** **2**). As shown in Figure 8, the anti‐bond orbital filling of Co—O_in_ bond in Co‐LDH@ZIF‐67 was significantly less than that in Co‐LDH (the blue area below Fermi level in Figure 8a,c), due to the strong interaction of the ligands with O_in_ and further confirmed the above charge analysis. Similarly, for the OER active site, it was clear that the Co—O_out_ bond of Co‐LDH@ZIF‐67 (–2.75) was stronger than that of Co‐LDH (–2.65), which made it easier to break the H—O_out_ bond in the PDS. Thus, the increased OER activity of Co‐LDH@ZIF‐67 was derived from the enhanced bonding strength of cobalt with oxygen at active site (Co—O_out_), which resulted in a lower free energy change of PDS (the dehydrogenation) in the OER process. Furthermore, the origin was the interaction of the inner ZIF‐67 with outer Co‐LDH, where the strong electron attraction of the ligand led to a decrease in the anti‐bond orbital filling of Co—O bond.

**Figure 8:**
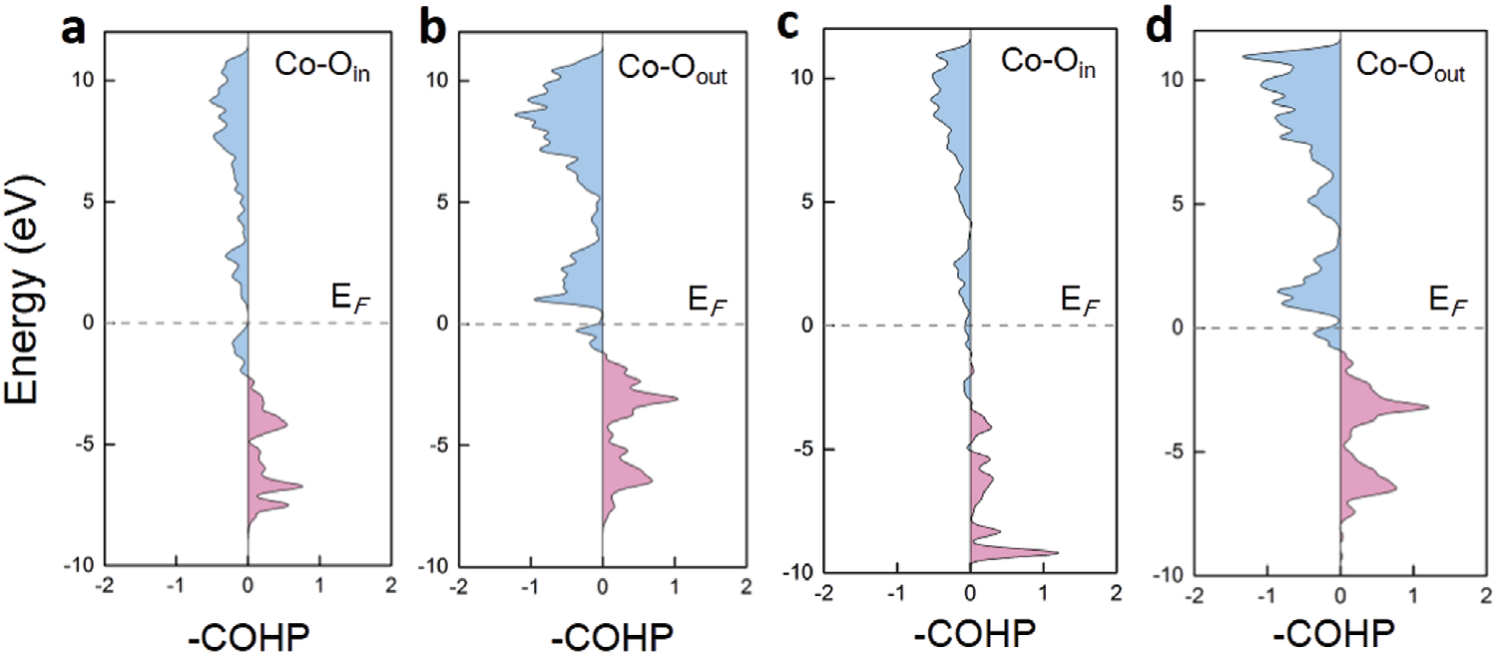
pCOHP for a) Co—O_in_ and b) Co—O_out_ in Co‐LDH, and for c) Co—O_in_ and d) Co—O_out_ in Co‐LDH@ZIF‐67. The positive represents the bonding contributions, and the negative represents the antibonding contributions.

**Table 1:** Calculated Bader charges, *q*, for local atoms of Co‐LDH and Co‐LDH@ZIF‐67

Local atoms	O_in_	Co_1_	Co_2_	Co_3_	O_out_
Co‐LDH	–1.23	1.55	1.53	1.53	–1.22
Co‐LDH@ZIF‐67	–0.66	1.37	1.51	1.50	–1.19

**Table 2:** The calculated ICOHP value of the investigated Co—O bond

ICOHP	Co_1_—O_in_	Co_1_—O_out_
Co‐LDH	–1.18	–2.65
Co‐LDH@ZIF‐67	–1.21	–2.75

In summary, we have developed a novel Co‐LDH@ZIF‐67 core–shell heterojunction structure using a simple aqueous solution‐based method solution using 2‐methylimidazole as the ligand and Co(NO_3_)_2_ as the cobalt precursor. The heterojunction interface was built by the O of Co‐LDH and N of the 2‐methylimidazole ligand in ZIF‐67. Impressively, the Co‐LDH@ZIF‐67 core–shell heterojunction structure exhibited higher electrocatalytic activity and stability toward OER under alkaline conditions than IrO_2_, Co‐LDH, and Co‐LDH/ZIF‐67, which was attributed to the interfacial interaction between ZIF‐67 and Co‐LDH and the large surface area and abundant porous structure of ZIF‐67. The Co‐LDH@ZIF‐67 exhibited an unprecedented overpotential of 187 mV at a current density of 10 mA cm^−2^ and remarkable electrochemical stability for more than 50 h. DFT calculations demonstrated that the interfacial interaction can enhance the strength of the Co—O_out_ bond in Co‐LDH, which made it easier to break the H—O_out_ bond and resulted in a lower free energy change of the PDS in the OER. This work provides a new strategy to improve the OER catalytic activity of LDH.

## Conflict of Interest

The authors declare no conflict of interest.

## Supporting information

Supporting Information
