# Supplementary material for: Interface Engineering of Co‐LDH@MOF Heterojunction in Highly Stable and Efficient Oxygen Evolution Reaction
Source: Adv Sci (Weinh). 2020 Nov 25;8(2):2002631. doi: 10.1002/advs.202002631 (PMC7816714; doi:10.1002/advs.202002631)
Supplement: Supplementary file 1 — Supporting Information [file ADVS-8-2002631-s001.pdf]

WILEY-VCH

© 2020 Wiley-VCH GmbH

ADVANCED SCIENCE
Open Access

Supporting Information

for *Adv. Sci.*, DOI: 10.1002/advs.202002631

Interface Engineering of Co-LDH@MOF Heterojunction in Highly Stable and Efficient Oxygen Evolution Reaction

Zhenxing Li, Xin Zhang, Yikun Kang, Cheng Cheng Yu, Yangyang Wen, Mingliang Hu, DongMeng*, Weiyu Song*, Yang Yang**

Supporting Information

Interface Engineering of Co-LDH@MOF Heterojunction in Highly Stable and Efficient Oxygen Evolution Reaction

Zhenxing Li[*], *Xin Zhang, Yikun Kang, Cheng Cheng Yu, Yangyang Wen, Mingliang Hu, DongMeng*[*], *Weiyu Song*[*], *Yang Yang*[*]

Prof. Z. Li[†], X. Zhang[†],C. Yu, Prof. Y. Wen, M. Hu
State Key Laboratory of Heavy Oil Processing, College of New Energy and Materials, China University of Petroleum (Beijing), Beijing 102249, China
Email: lizx@cup.edu.cn
Y. Kang, Prof. W. Song
College of Science, China University of Petroleum (Beijing), Beijing 102249, China
Email: songwy@cup.edu.cn

Prof. D. Meng，Prof. Y. Yang

Department of Materials Science and Engineering, and California Nano Systems Institute, University of California, Los Angeles, CA, 90095, United States.
Email: dongmeng2017@ucla.edu, yangy@ucla.edu
[†]: These authors contributed equally to this work.

Experimental Section

Preparation of ZIF-67

In a typical synthetic procedure, $Co(NO_3)_2$ (0.0515 mmol) and 2-Methylimidazole (3.37 mmol) with Hexadecyl trimethyl ammonium Bromide (CTAB) (0.00548 mmol) were dispersed into deionized water (8 mL), respectively; subsequently, $Co(NO_3)_2$ solution was poured into 2-Methylimidazole solution with stirring. The mixture was allowed to stir for 24 hours at 25 ℃. The final dark violet product was obtained through centrifuging and washing procedures (with deionized water for twice and ethanol for once). Then, it was dried in vacuum at 60 ℃ for 12 hours before further characterization.

Preparation of Co-LDH@ZIF-67

$Co(NO_3)_2$ (0.0515 mmol) and 2-Methylimidazole (3.37 mmol) with Hexadecyl trimethyl ammonium Bromide (CTAB) (0.00548 mmol) were dispersed into deionized water (8 mL), respectively; subsequently, $Co(NO_3)_2$ solution was poured into 2-Methylimidazole solution with stirring. The mixture was allowed to stir for 20 min at 25 ℃. The product was obtained through centrifuging and washing procedures (with deionized water for twice and ethanol for once). Then, it was dried in vacuum at 60 ℃ for 12 hours before further characterization.

Preparation of Co-LDH

$Co(NO_3)_2$ (0.0515 mmol) and 2-Methylimidazole (3.37 mmol) with Hexadecyl trimethyl ammonium Bromide (CTAB) (0.00548 mmol) were dispersed into deionized water (8 mL), respectively; subsequently, $Co(NO_3)_2$ solution was poured into 2-Methylimidazole solution with stirring. The mixture was allowed to stir for 20 min at 25 ℃. The product was obtained through centrifuging, ultrasound (5 min each time) and washing procedures (with deionized water for three times and ethanol for once). Then, it was dried in vacuum at 60 ℃ for 12 hours before further characterization.

Preparation of Co-LDH/ZIF-67

Co-LDH (1.6mg) and 2-Methylimidazole (1.22 mmol) were dispersed into deionized water (5 mL), respectively, subsequently, 2-Methylimidazole solution was poured into Co-LDH solution with stirring. The mixture was allowed to stir for 6 hours at room temperature. The product was obtained through centrifuging and washing procedures (with deionized water for twice and ethanol for once). Then, it was dried in vacuum at 60 ℃ for 12 hours before further characterization.

Characterization

The morphology of as-prepared samples was characterized by scanning electron microscopy (SEM, Hitachi SU8010) and transmission electron microscopy (TEM, JEM 2100 LaB6). Powder X-ray diffractometer (XRD) analysis was conducted on a Bruker D8 Advance instrument with a Cu Kα irradiation source at a scanning rate of 5° per min. Raman spectroscopy was performed on a HORIBA labRAM HR Evolution with a 633 nm excitation wavelength. Fourier transform infrared (FTIR) spectra were taken on a Bruker VERTEX 70 instrument. UV-Vis absorption spectra were investigated on a Hitachi U-3010 spectrometer. X-ray photoelectron spectroscopy (XPS) was carried out on a PHI5000 Versaprobe using an Al Kα X-ray source, with all the binding energies calibrated to C 1s peak at 285.0 eV. The specific surface areas and pore size distribution of samples were evaluated on the ASAP2460 Surface Area and Porosity Analyzer (Micromeritics), calculated by N_2 sorption isotherms using a Brunauer-Emmett-Teller (BET) method. The atomic force microscopy (AFM) image was measured at a Bruker Scan-Dimension-Icon system. Thermal gravimetric analysis (TGA) was characterized on Mettler Toledo TGA/DSC 3+ thermogravimetric analyzer under argon or air atmosphere at a heating rate of 10 °C min^{-1} in the temperature range of 30-800 °C. ICP-MS analysis was conducted by an Agilent 7500cx instrument.

Electrochemical measurements

All the electrochemical experiments were performed in a CHI 660E potentiostat (Shanghai Chenhua Instrument Factory, China). A conventional three-electrode system was

applied using the graphite rod and Ag/AgCl (3 M KCl) as the counter and reference electrode, respectively. The working electrode was prepared with the following procedures. Firstly, the well-dispersed catalyst ink was prepared by dispersing 2.0 mg of catalyst and 0.2 mg of carbon black in 400 µL of solution containing 100 µL of ethanol, 390 µL of water and 10 µL of 5wt% nafion solution. Then, 20 µL of uniform solution was dripped onto the carbon cloth (l=0.5 cm, S=0.25 cm^2), and dried at room temperature with a catalyst loading of 400 µg cm^{-2}. For IrO_2, the well-dispersed catalyst ink was prepared by dispersing 2.0 mg of IrO_2 and 0.2 mg of carbon black in 400 µL of solution containing 100 µL of ethanol, 390 µL of water and 10 µL of 5 wt% nafion solution. Then, 20 µL of uniform solution was dripped onto the carbon cloth (l=0.5 cm, S=0.25 cm^2), and dried at room temperature with a catalyst loading of 400 µg cm^{-2}. Before evaluating the OER activity, O_2 gas was bubbled through the 1 M KOH solution for 30 min to obtain O_2 saturated solution. Linear sweep voltammograms (LSV) were measured at a scan rate of 5 mV s^{-1} with 80% iR compensation were converted to reverse hydrogen electrode (RHE). Cyclic voltammograms were recorded at various scan rates (10, 20, 40, 60, 80, and 100 mV s^{-1}) to estimate the double-layer capacitance. All the potentials were calibrated to reversible hydrogen electrode (RHE). EIS was performed at 0.55 V over a frequency range from 100 mHz to 10 kHz with an amplitude of 5 mV. The durability test was assessed at 30mA cm^{-1} over a 50 hours period. After the electrochemical test, the Fe content in the electrolyte was analyzed by ICP-MS, and no Fe was detected. For comparison, the commercial IrO_2 catalysts (Alfa Aesar) were also tested with the same experiment conditions.

Computational details.

Density functional theory (DFT) calculations were performed using Vienna ab initio Simulation Package (VASP)[1-2] with the generalized gradient approximation (GGA) parameterized by Perdew, Burke and Ernzerhof (PBE) for the exchange correlation functional.[3] We used a two layer p (2 × 2) slab of $Co(OH)_2$ (001) slab and fixed the bottom layer to simulate Co-LDH. And we used a one layer p (2 × 2) slab of $Co(OH)_2$ (001) slab with

two lower ZIF-67 ligands to simulate Co-LDH@ZIF-67. An energy cutoff of 400 eV is used for all calculations, and the k-point meshes of 3 × 2 × 1 were used for Brillouin zone integration. The atomic positions were relaxed until the force on each atom was less than 0.05 eV/Å, and the convergence tolerance of the energy was set to be 10^{-5} eV.

A simple rotationally invariant DFT+U version was used to take into account the electronic correlation of Co 3d electrons. We used a value of U = 3.3 eV for Co atoms, which is the same as Deng et al.[4]

The OER process is generally considered to be a four-step electron transfer mechanism:[5]

$OH^- + * \rightarrow OH^* + e^-$ (1)

$OH^* + OH^- \rightarrow O^* + H_2O + e^-$ (2)

$O^* + OH^- \rightarrow OOH^* + e^-$ (3)

$OOH^* \rightarrow O_2 + H_2O + e^-$ (4)

where * and i* represent the active site and the adsorbed intermediate on the surface, respectively.

The Gibbs free energy differences of these intermediates including zero point energy (ZPE) and entropy corrections (TΔS) can be calculated as

$\Delta G_i = \Delta E_i + \Delta ZPE_i - T\Delta S_i$ (5)

where energy differences ΔE_i is calculated as follows:

$\Delta E_{OH} = E(OH^*) - E(*) - [E(H_2O) - 1/2E(H_2)]$ (6)

$\Delta E_O = E(O^*) - E(*) - [E(H_2O) - E(H_2)]$ (7)

$\Delta E_{OOH} = E(OOH^*) - E(*) - [2E(H_2O) - 3/2E(H_2)]$ (8)

The Gibbs free energy change for steps (5) - (8) can be expressed as

$\Delta G_1 = \Delta G_{OH} - eU$ (9)

$\Delta G_2 = \Delta G_O - \Delta G_{OH} - eU$ (10)

$\Delta G_3 = \Delta G_{OOH} - \Delta G_O - eU$ (11)

$\Delta G_4 = 4.92 - \Delta G_{OOH} - eU$ (12)

where U is the potential measured against normal hydrogen electrode (NHE) at standard conditions.

The catalytic performance was estimated by the free energy (G^{OER}) of potential-determining step (PDS) in the OER process:

$$G^{OER} = \max [\Delta G_1, \Delta G_2, \Delta G_3, \Delta G_4] \quad (13)$$

As commonly practiced, the standard chemical potential of H_2O (l) is equivalent to the DFT total energy of H_2O(g) together with corrections for the zero-point energy (ZPE) and entropy at 25°C and 0.035 atm. Through our tests, the ZPE difference of the same species adsorbed at Co-LDH and Co-LDH@ZIF-67 is less than 0.02 eV, so we use the following ZPE uniformly.

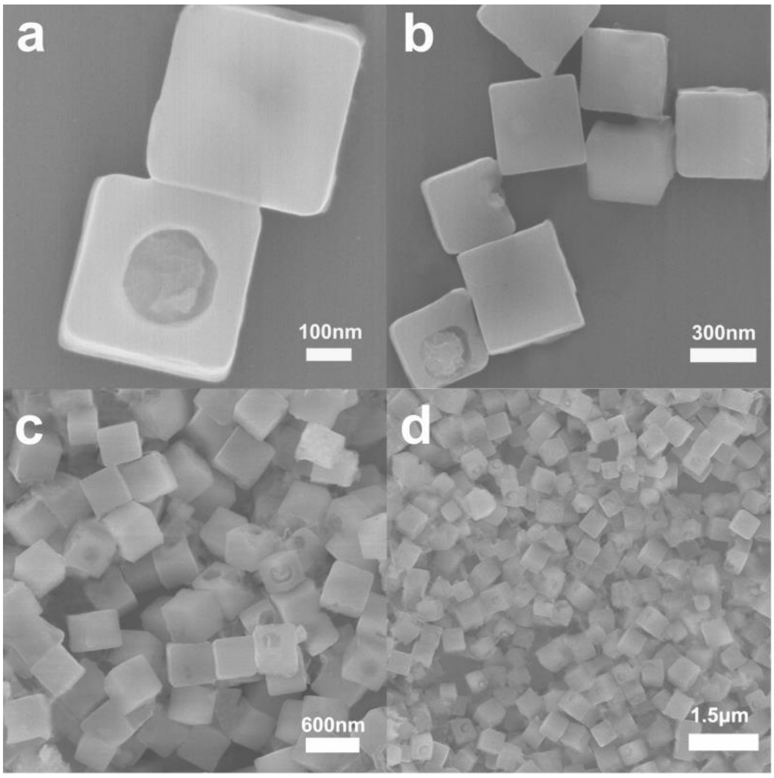

Figure S1. SEM image of Co-LDH@ZIF-67.

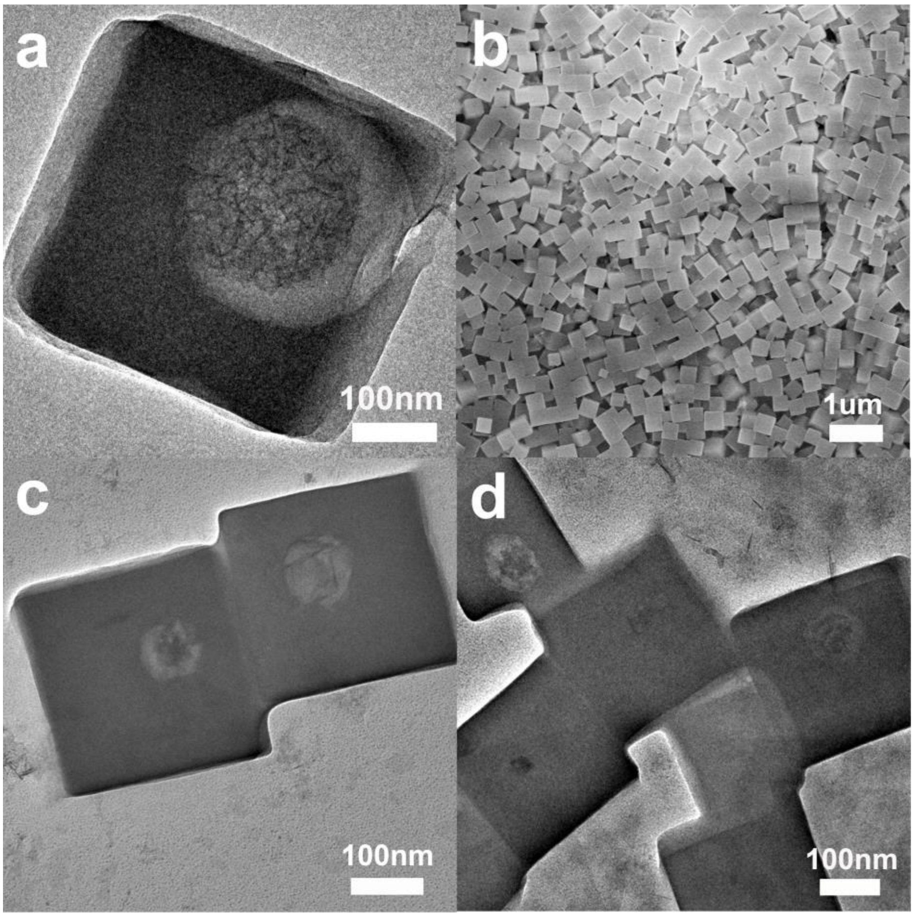

Figure S2. TEM images of the samples obtained in different time a) 20min. c,d) 6h. b) SEM image of the sample obtained in 24 hours.

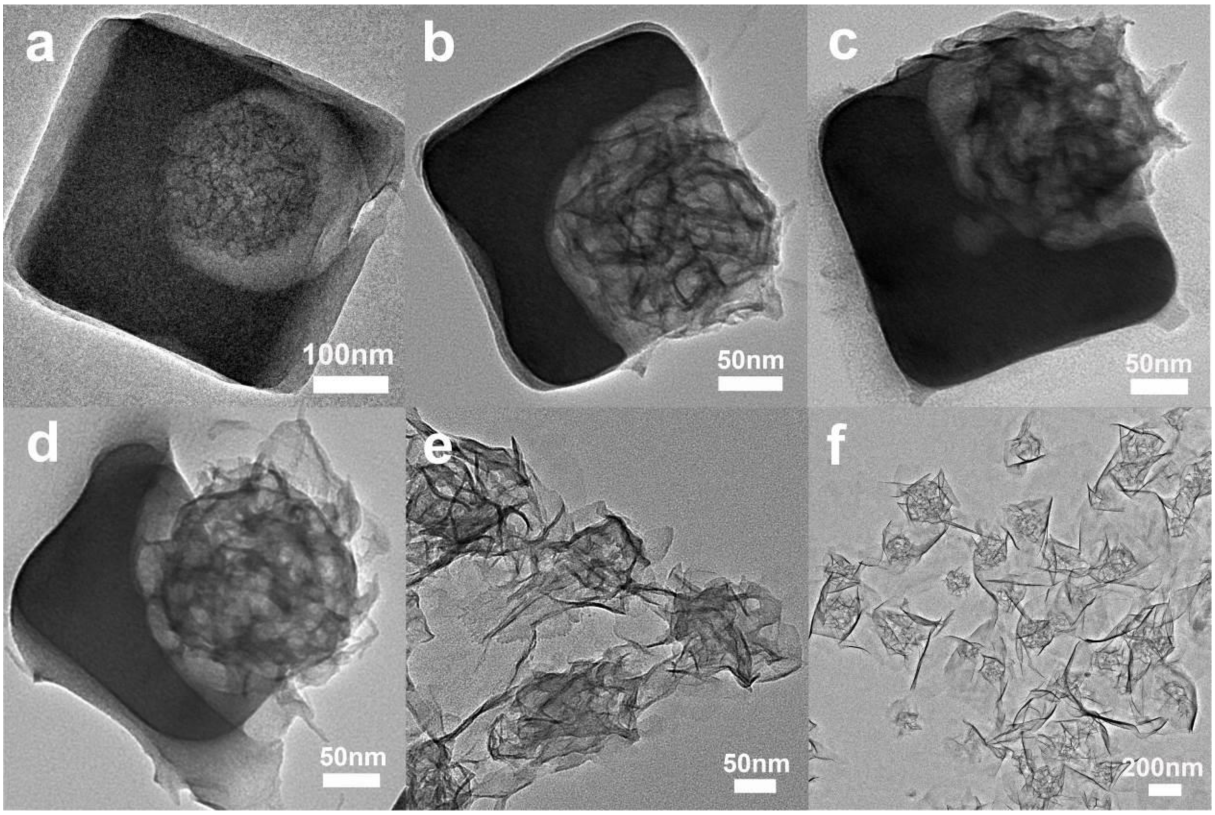

Figure S3. TEM images of the samples with different ultrasound times a) 0 min. b,c) 5 min. d) 10min. e,f) 15min.

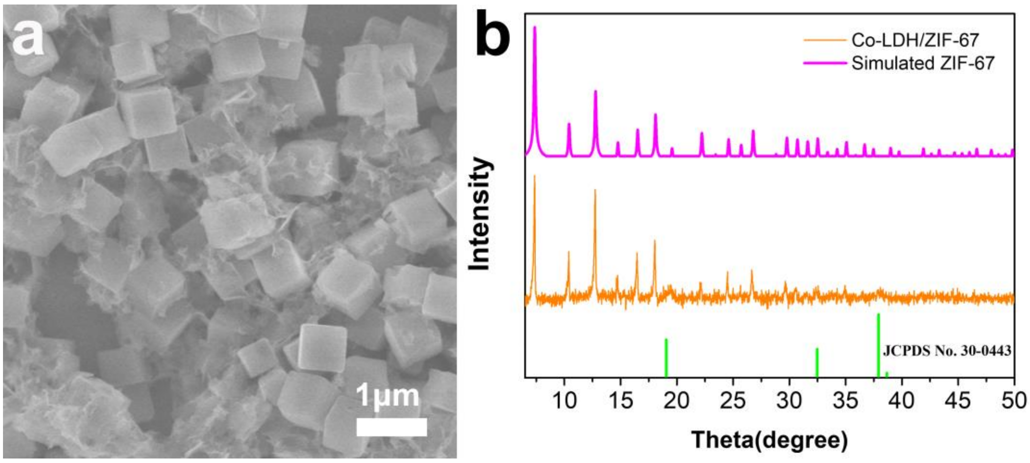

Figure S4. a)SEM image of Co-LDH/ZIF-67. b) XRD pattern of Co-LDH/ZIF-67.

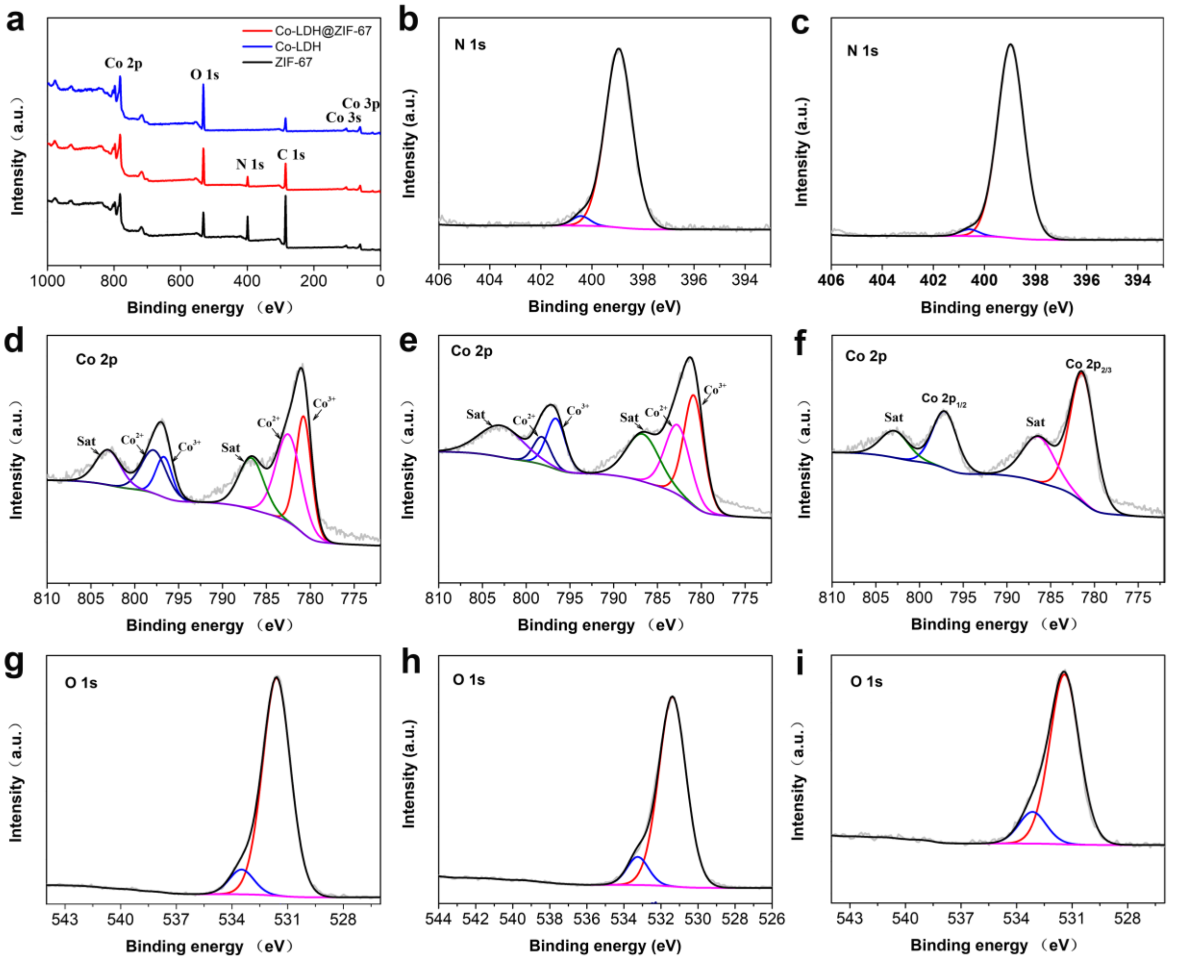

Figure S5. a) XPS wide scan spectra of Co-LDH@ZIF-67, Co-LDH and ZIF-67. d, g) High-resolution Co 2p XPS spectrum and high-resolution O 1S XPS spectrum of Co-LDH. b, e, h) High-resolution N 1S XPS spectrum, high-resolution Co 2p XPS spectrum and high-resolution O 1S XPS spectrum of Co-LDH@ZIF-67. c, f, i) High-resolution N 1S XPS spectrum , high-resolution Co 2p XPS spectrum and high-resolution O 1S XPS spectrum of ZIF-67.

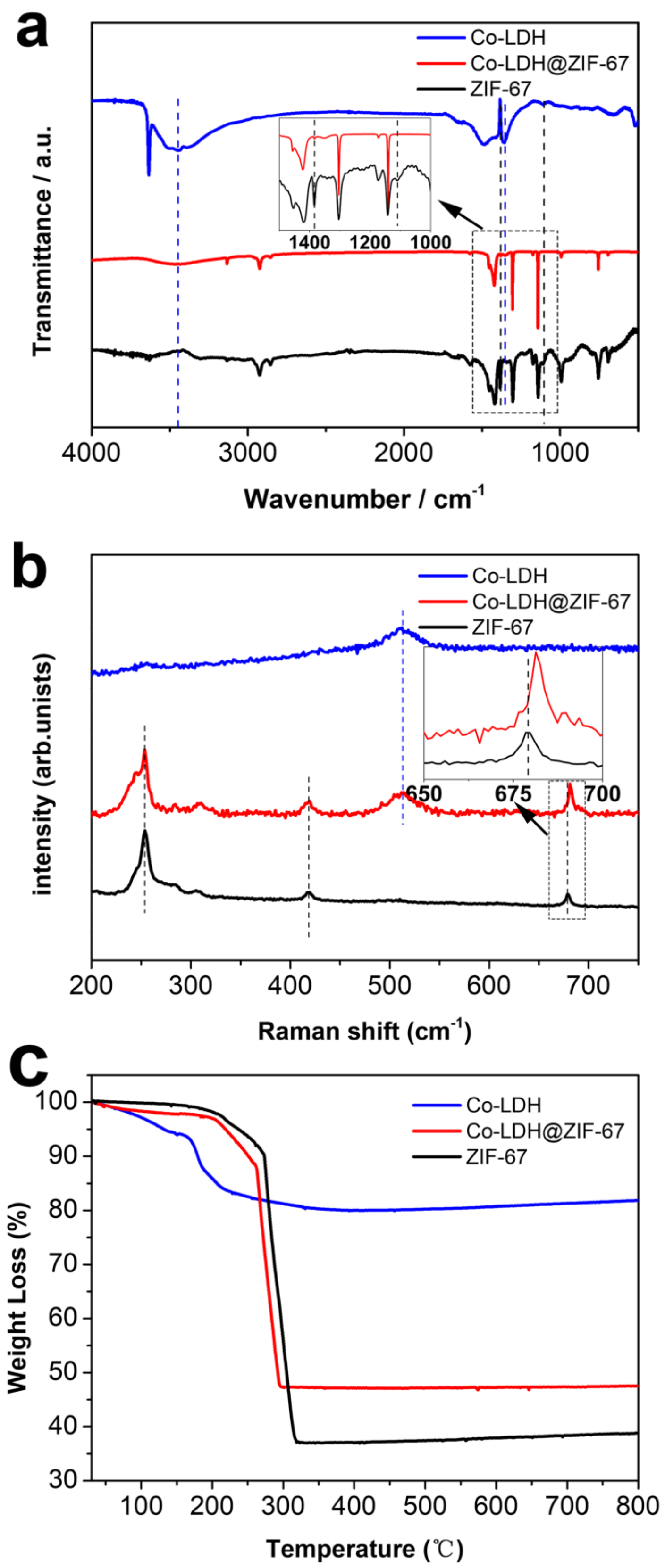

Figure S6. a) FTIR spectrum, b) Raman spectra, and c) TGA curve under air atmosphere of Co-LDH@ZIF-67, Co-LDH and ZIF-67.

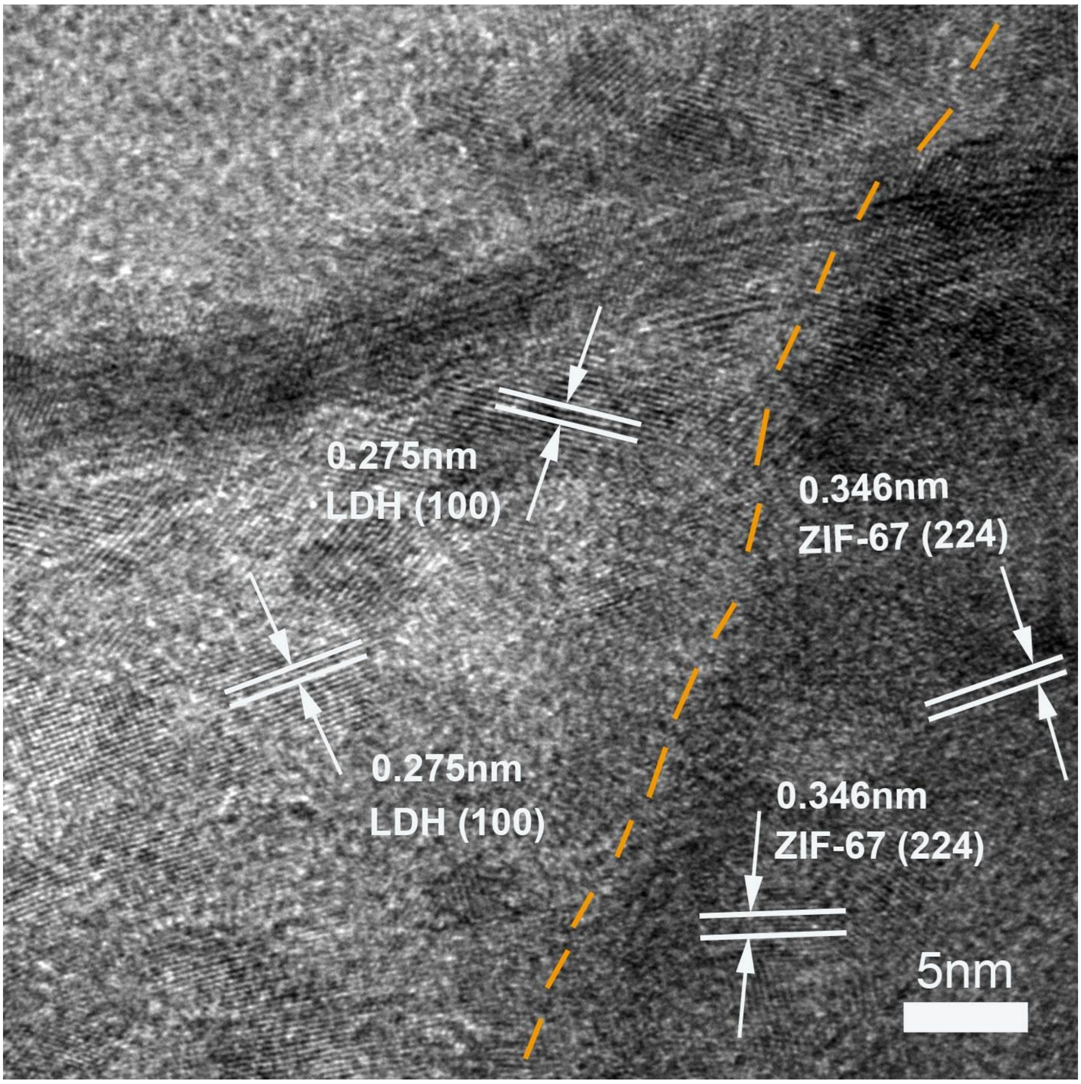

Figure S7. HRTEM image of Co-LDH@ZIF-67.

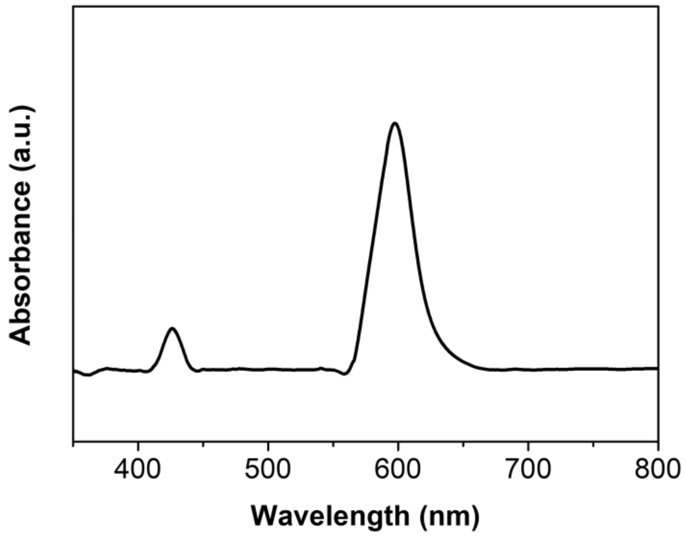

Figure S8. The UV-vis spectrum of Co-LDH@ZIF-67.

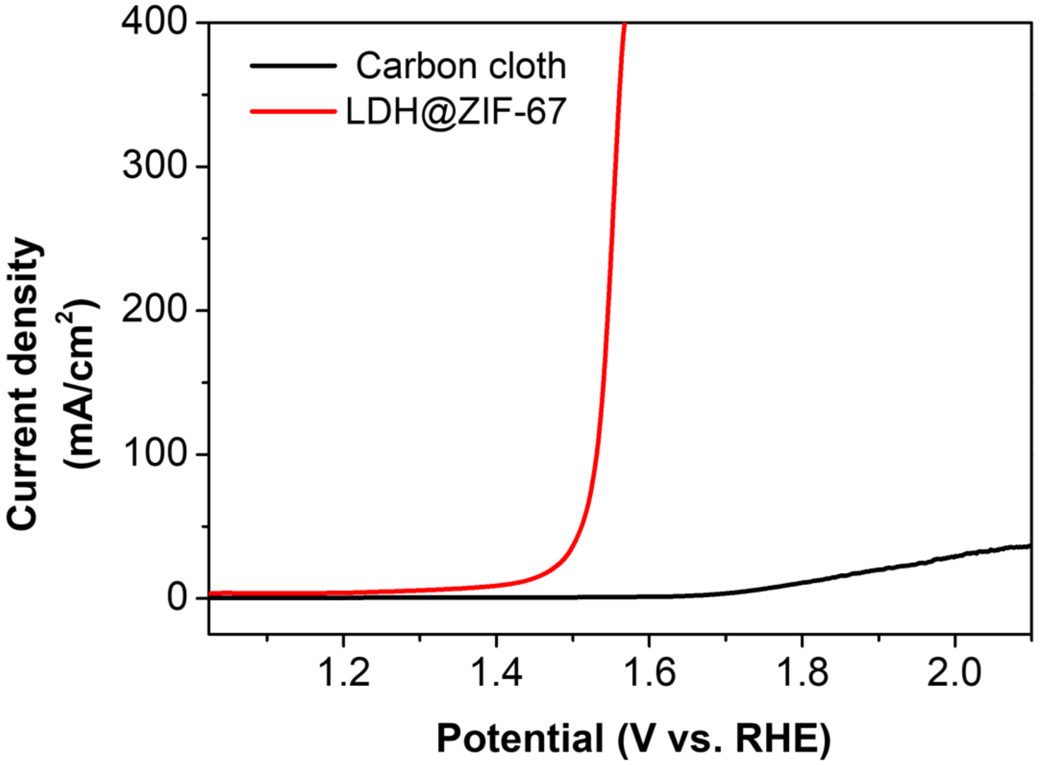

Figure S9. OER polarization curve of Co-LDH@ZIF-67 and carbon cloth.

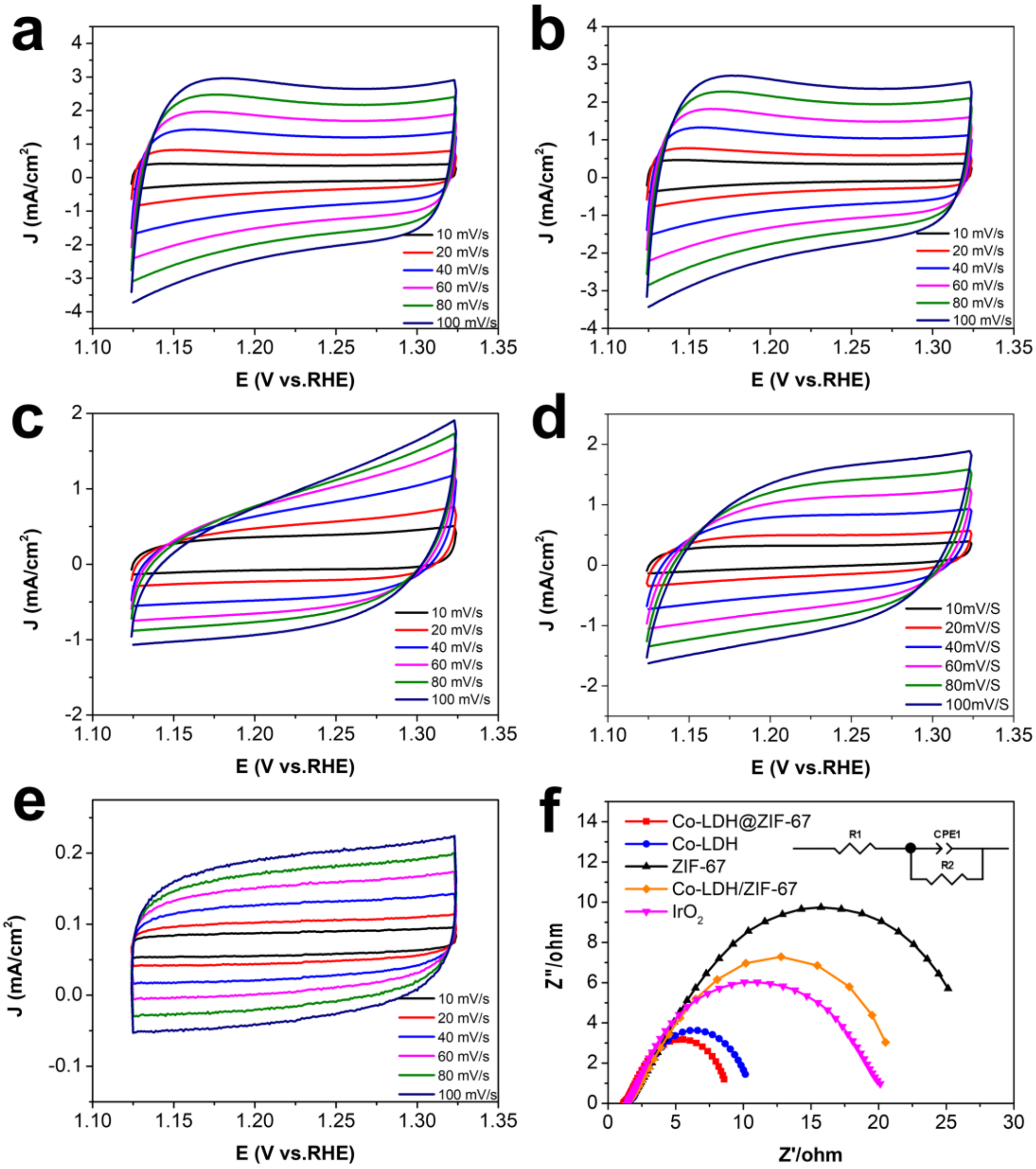

Figure S10. CV of a) Co-LDH@ZIF-67. b) Co-LDH. c) ZIF-67 d) Co-LDH/ZIF-67 and e) IrO_2. f) The Nyquist plots at the overpotential of 344 mV of Co-LDH@ZIF-67, Co-LDH, ZIF-67, Co-LDH/ZIF-67 and IrO_2. upper right inset: equivalent circuit model (R1: electrolyte resistance, CPE1: double layer capacity, R2: charge transfer resistance).

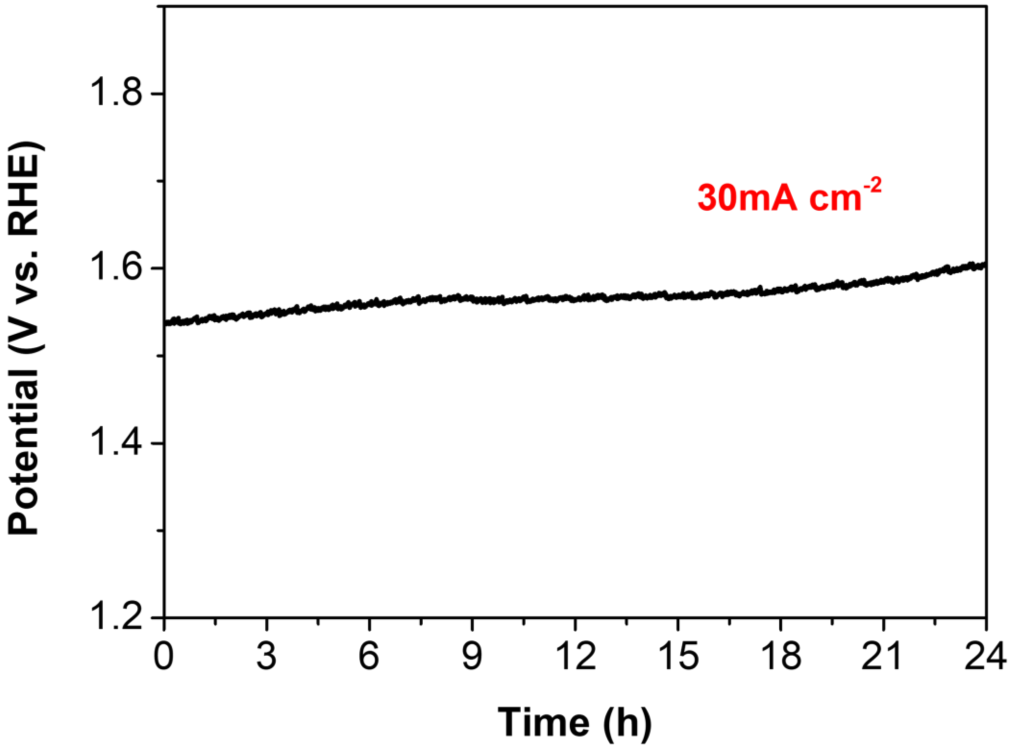

Figure S11. Long-term stability test of Co-LDH carried out under a constant current density of 30 mA cm^{-2}.

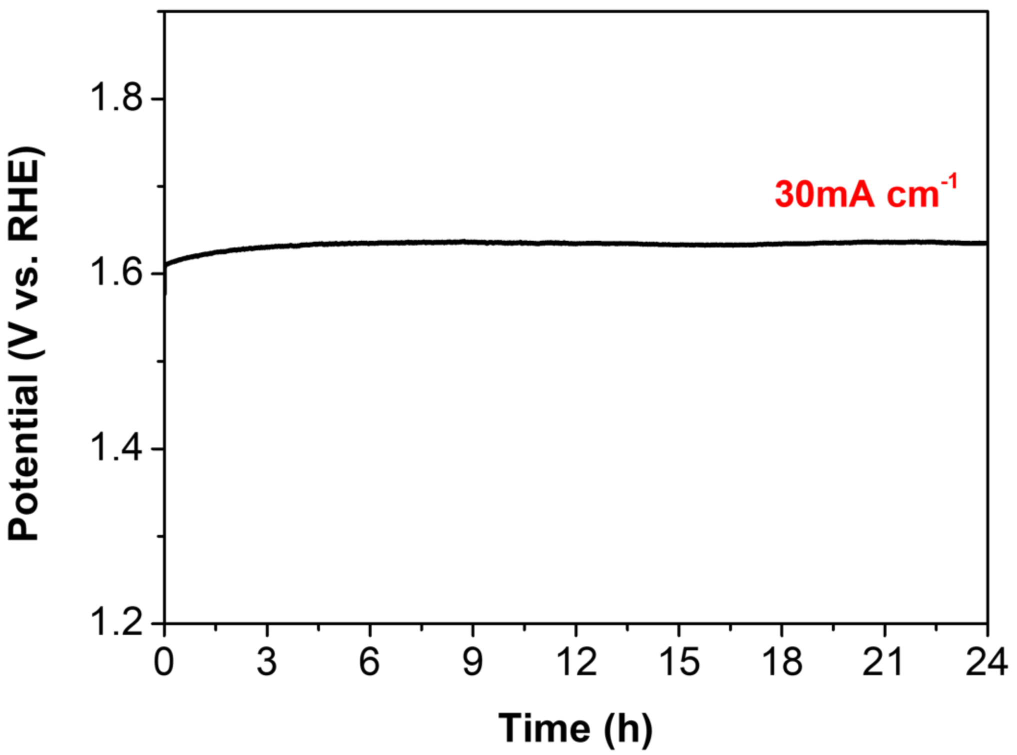

Figure S12. Long-term stability test of ZIF-67 carried out under a constant current density of 30 mA cm^{-2}.

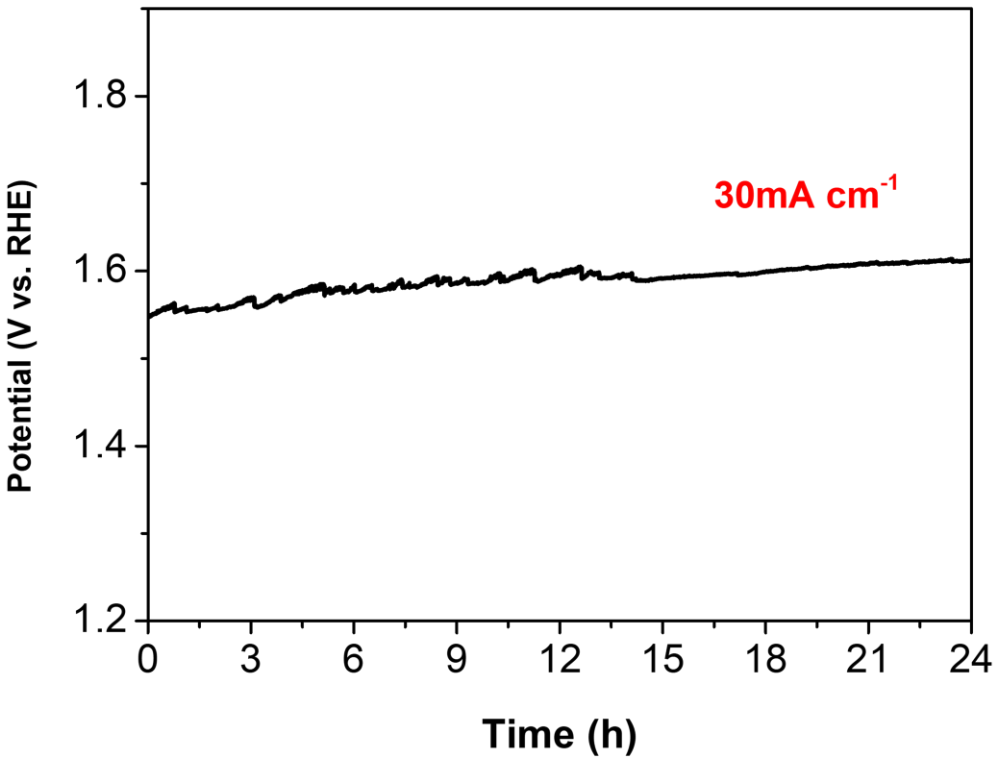

Figure S13. Long-term stability test of Co-LDH/ZIF-67 carried out under a constant current density of 30 mA cm^{-2}.

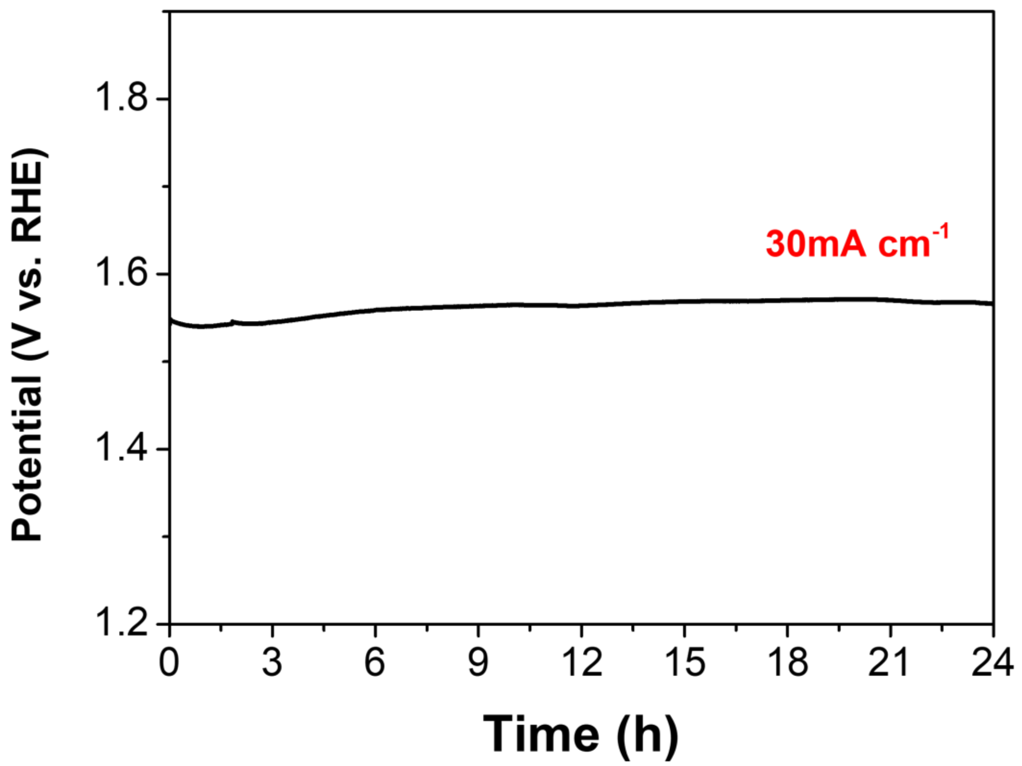

Figure S14. Long-term stability test of IrO_2 carried out under a constant current density of 30 mA cm^{-2}.

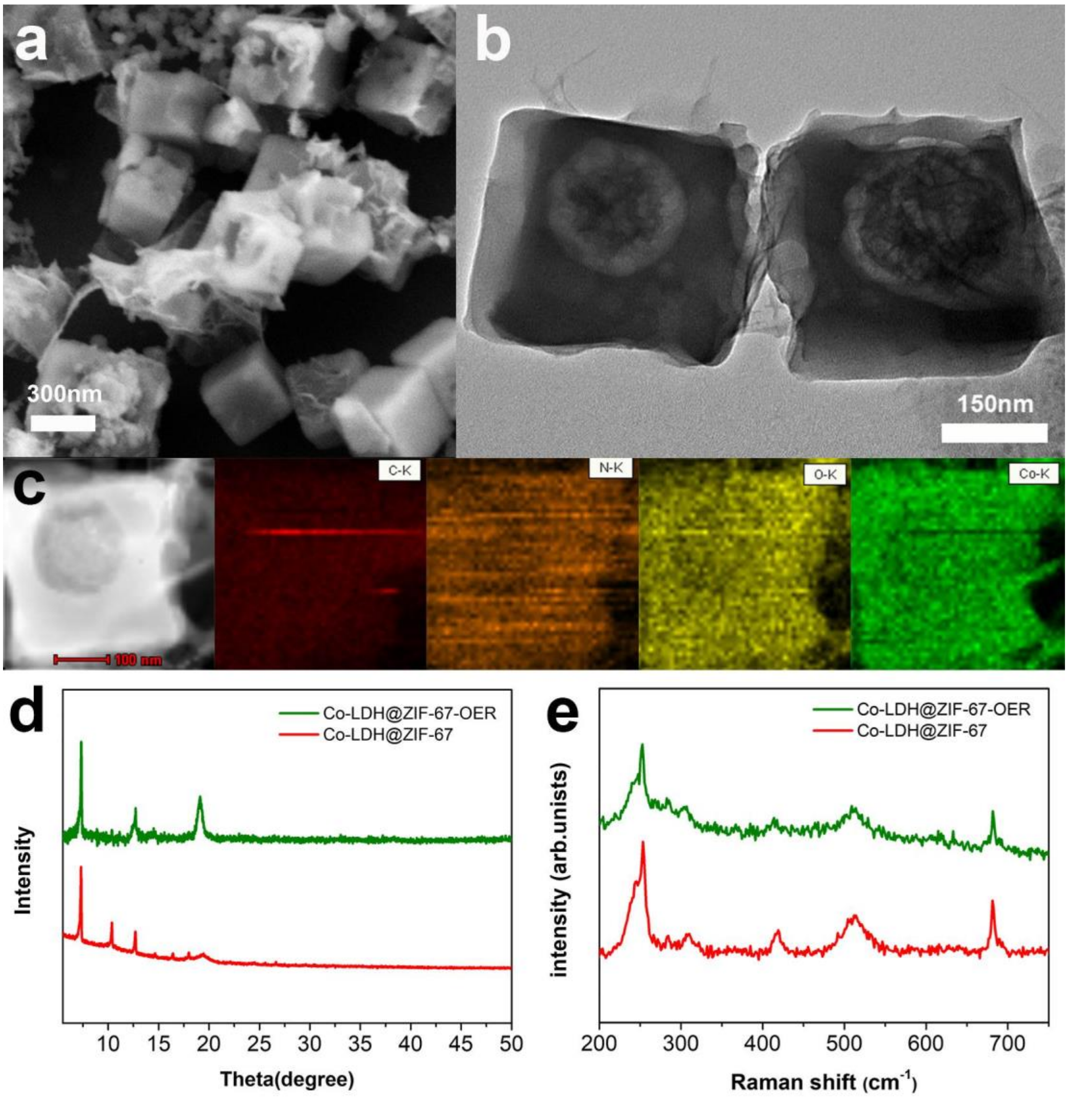

Figure S15. a) SEM images, b) TEM image and c) EDX elemental mappings of Co-LDH@ZIF-67 after OER process. d) XRD pattern and e) Raman spectra of Co-LDH@ZIF-67 and Co-LDH@ZIF-67 after OER process.

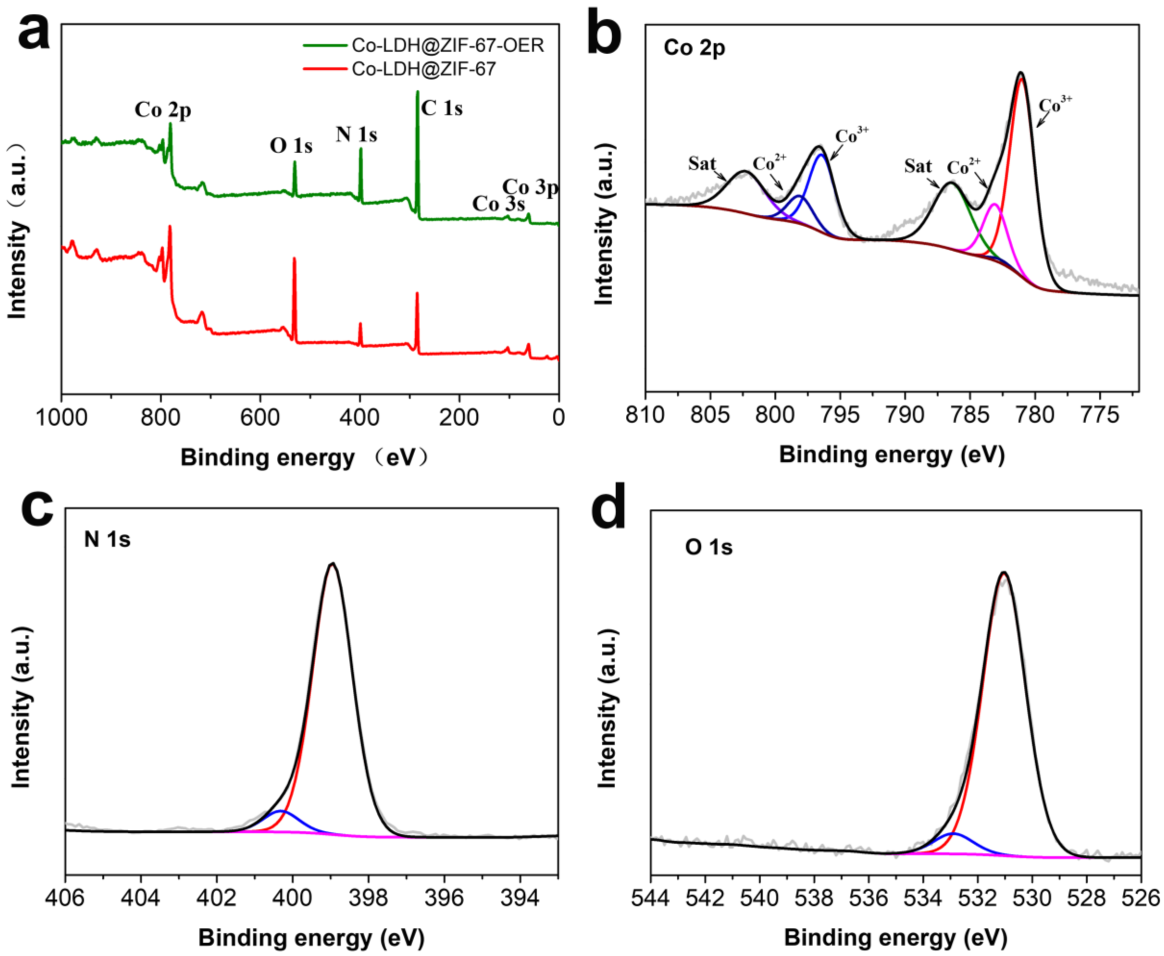

Figure S16. a) XPS wide scan spectra of Co-LDH@ZIF-67 and Co-LDH@ZIF-67 after OER process. b) high-resolution Co 2p XPS spectrum. c) high-resolution N 1S XPS spectrum and d) high-resolution O 1S XPS spectrum of the Co-LDH@ZIF-67 after OER process.

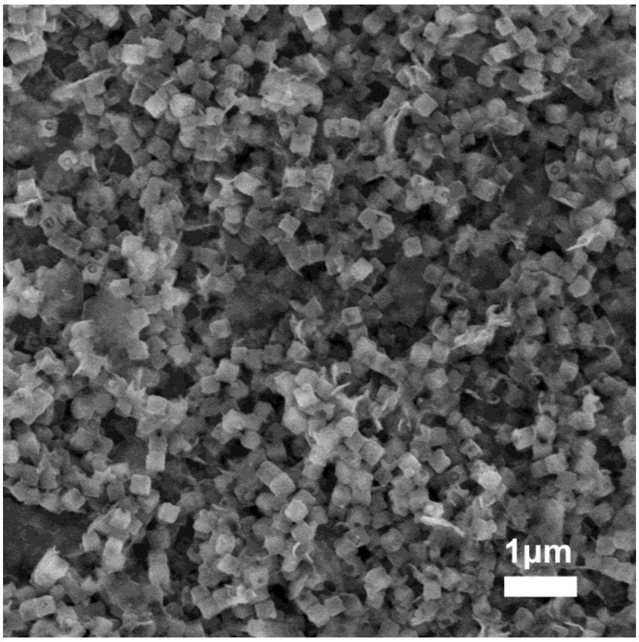

Figure S17. SEM images of Co-LDH@ZIF-67 that deposited onto the working electrode and placed in 1 M KOH solution for 24 hours.

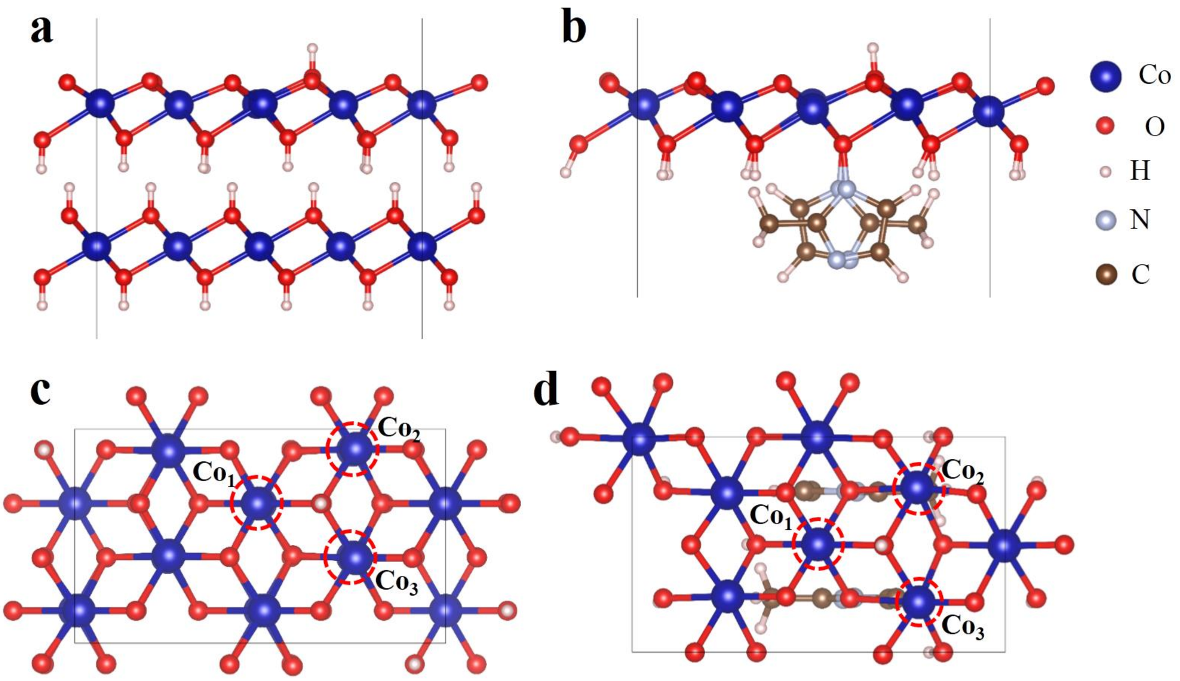

Figure S18. The side view for the optimized slab models of a) Co-LDH and (b) Co-LDH@ZIF-67, and the top view of c) Co-LDH and d) Co-LDH@ZIF-67.

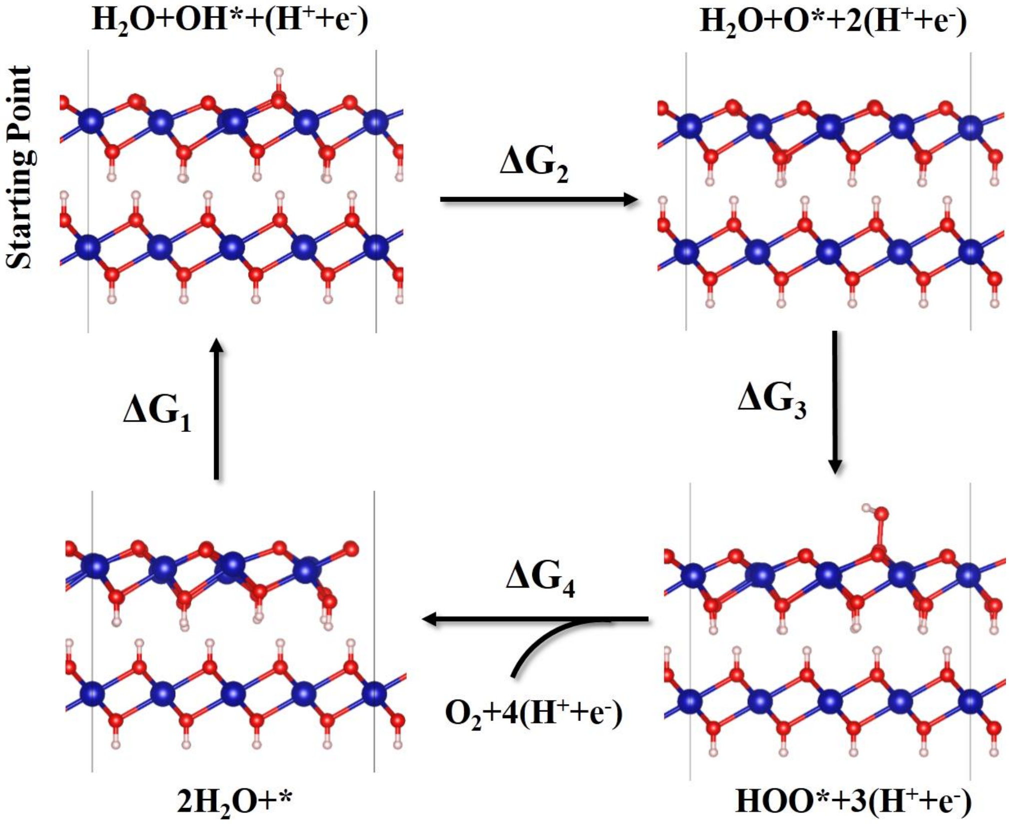

Figure S19. Schematic diagram of the four-step OER on Co-LDH.

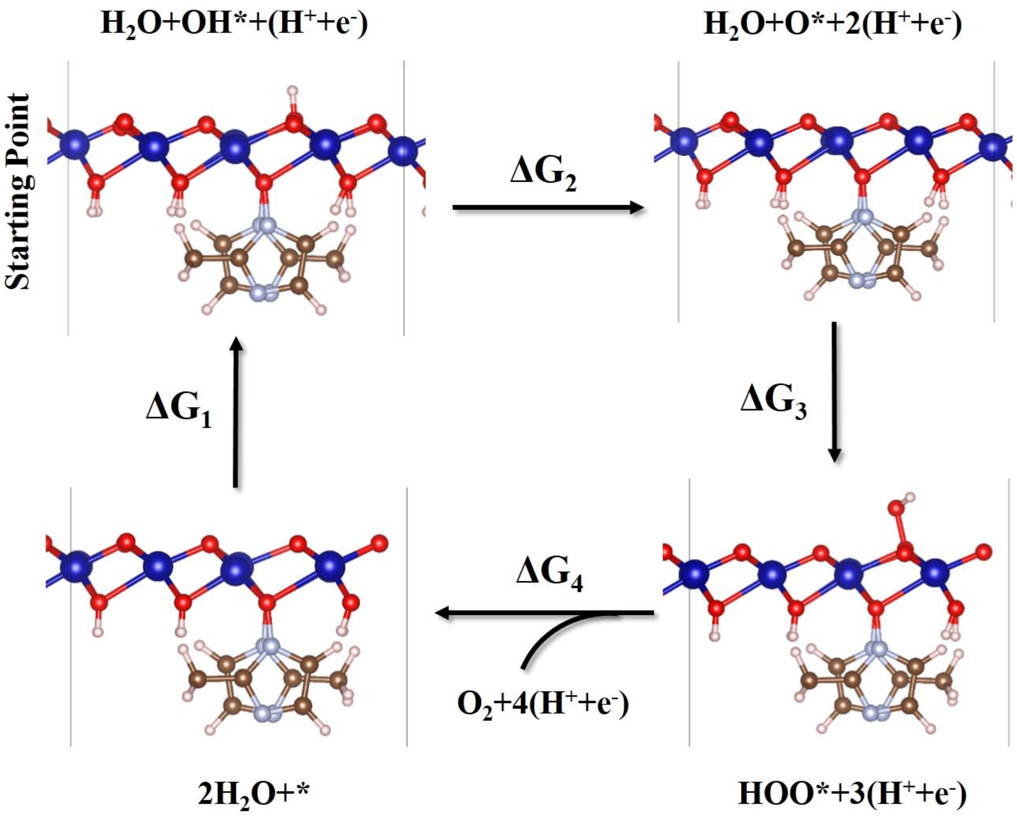

Figure S20. Schematic diagram of the four-step OER on Co-LDH@ZIF-67.

Table S1. Comparison of the OER performance of Co-LDH@ZIF-67 with other reported OER electrocatalysts in basic electrolyte.

Electrocatalysts	Electrolyte	Overpotential vs. 10 mA cm^{-2}	Ref
Co_3O_4/CeO_2	1M KOH	270 mV	6
$(Ni_2Co_1)_{0.925}Fe_{0.075}$-MOF-NF	1M KOH	257 mV	7
Co-Fe double-atom	1M KOH	321 mV	8
PM-LDH	1M KOH	230 mV	9
Co@N-CS/N-HCP@CC	1M KOH	248mV	10
Co–N_x/C NRA	1M KOH	300mV	11
HCM@Ni-N	1M KOH	304 mV	12
MoS_2/FNS/FeNi	1M KOH	204 mV	13
NiFe LDH@NiCoP/NF	1M KOH	220 mV	14
Ni_3N-VN/NF || Ni_2P-VP_2/NF	1M KOH	280 mV	15
Co_3O_4/HNCP-40	1M KOH	333 mV	16
CoFe-MOF-OH	1M KOH	265 mV	17
CoNi-LDH@ PCPs	1M KOH	350 mV	18
NiCo/Fe_3O_4/MOF-74	1M KOH	238 mV	19
Au/NiFe LDH	1M KOH	237 mV	20
Fe^{2+}-NiFe LDH	1M KOH	195 mV	21
**Co-LDH@ZIF-67**	**1M KOH**	**187 mV**	**This work**

Table S2. Corrections for zero-point energy and entropy (T = 298.15 K).

	TS	ZPE
H_2O (l)	0.67	0.57
OH*	0	0.38
O*	0	0.07
OOH*	0	0.45

Table S3. EIS data obtained by fitting the experimental data in Figure S9f.

Electrocatalysts	R1 (Ω)	R2 (Ω)
Co-LDH@ZIF-67	1.718	7.336
Co-LDH	1.867	9.03
ZIF-67	1.492	28.59
Co-LDH/ZIF-67	1.964	20.36
IrO_2	1.641	18.7

Reference

[1] G. Kresse, J. Furthmuller, *Phys. Rev. B: Condens. Matter* **1996**, *54*,11169.
[2] G. Kresse, J. Furthmuller, *Comp. Mater. Sci.* **1996**, *6*, 15.
[3] J. Perdew , K. Burke, M. Ernzerhof, *Phys. Rev. Lett.* **1996**, *77*, 3865.
[4] T. Deng, W. Zhang, O. Arcelus, J. G. Kim, J. Carrasco, S. J. Yoo, W. Zheng, J. Wang, H. Tian, H. Zhang, X. Cui, T. Rojo, *Nat. Commun.* **2017**, *8*, 15194.
[5] L. Peng, J. Wang, Y. Nie, K. Xiong, Y. Wang, L. Zhang, K. Chen, W. Ding, L. Li, Z. Wei, *ACS Catal.* **2017**, *7*, 8184.
[6] Y. Liu, C. Ma, Q. Zhang, W. Wang, P. Pan, L. Gu, D. Xu, J. Bao, Z. Dai, *Adv. Mater.* **2019**, *31*, 1900062.
[7] Q. Qian, Y. Li, Y. Liu, L. Yu, G. Zhang, *Adv. Mater.* **2019**, *31*, 1901139.
[8] L. Bai, C. S. Hsu, D. T. L. Alexander, H. M. Chen, X. Hu, *J. Am. Chem. Soc.* **2019**, *141*, 14190.
[9] X. Zhang, Y. Zhao, Y. Zhao, R. Shi, G. I. N. Waterhouse, T. Zhang, *Adv. Energy Mater.* **2019**, 9, 1900881.
[10] Z. Chen, Y. Ha, H. Jia, X. Yan, M. Chen, M. Liu, R. Wu, *Adv. Energy Mater.* **2019**, *9*, 1803918.
[11] I. S. Amiinu, X. Liu, Z. Pu, W. Li, Q. Li, J. Zhang, H. Tang, H. Zhang, *S. Mu, Adv. Funct. Mater.* **2018**, *28*, 1704638.
[12] H. Zhang, Y. Liu, T. Chen, J. Zhang, J. Zhang, X. W. D. Lou, *Adv. Mater.* **2019**, *31*, 1904548.
[13] Y. Wu, F. Li, W. Chen, Q. Xiang, Y. Ma, H. Zhu, P. Tao, C. Song, W. Shang, T. Deng, J. Wu, *Adv. Mater.* **2018**, *30*, 1803151.
[14] H. Zhang, X. Li, A. Hähnel, V. Naumann, C. Lin, S. Azimi, S. L. Schweizer, A. W. Maijenburg, R. B. Wehrspohn, *Adv. Funct. Mater.* **2018**, *28*, 1706847.
[15] H. Yan, Y. Xie, A. Wu, Z. Cai, L. Wang, C. Tian, X. Zhang, H. Fu, *Adv. Mater.* **2019**, *31*, 1901174.
[16] D. Ding, K. Shen, X. Chen, H. Chen, J. Chen, T. Fan, R. Wu, Y. Li, *ACS Catal.* **2018**, *8*, 7879.
[17] Z. Zou, T. Wang, X. Zhao, W.-J. Jiang, H. Pan, D. Gao, C. Xu, *ACS Catal.* **2019**, *9*, 7356.
[18] W. Wang, Y. Lu, M. Zhao, R. Luo, Y. Yang, T. Peng, H. Yan, X. Liu, Y. Luo, *ACS nano* **2019**, *13*, 12206.
[19] X. Wang, H. Xiao, A. Li, Z. Li, S. Liu, Q. Zhang, Y. Gong, L. Zheng, Y. Zhu, C. Chen, D. Wang, Q. Peng, L. Gu, X. Han, J. Li, Y. Li, *J. Am. Chem. Soc.* 2018, 140, 15336.
[20] J. Zhang, J. Liu, L. Xi, Y. Yu, N. Chen, S. Sun, W. Wang, K. M. Lange, B. Zhang, *J. Am. Chem. Soc.* **2018**, *140*, 3876.
[21] Z. Cai, D. Zhou, M. Wang, S. M. Bak, Y. Wu, Z. Wu, Y. Tian, X. Xiong, Y. Li, W. Liu, S. Siahrostami, Y. Kuang, X. Q. Yang, H. Duan, Z. Feng, H. Wang, X. Sun, *Angew. Chem. Int. Ed.* **2018**, *57*, 9392.
